# The Academic Backbone: longitudinal continuities in educational achievement from secondary school and medical school to MRCP(UK) and the specialist register in UK medical students and doctors

**DOI:** 10.1186/1741-7015-11-242

**Published:** 2013-11-14

**Authors:** IC McManus, Katherine Woolf, Jane Dacre, Elisabeth Paice, Chris Dewberry

**Affiliations:** 1 UCL Medical School, University College London, Gower Street, London WC1E 6BT, UK; 2 Research Department of Clinical, Educational and Health Psychology, Division of Psychology and Language Sciences, University College London, Gower Street, London WC1E 6BT, UK; 3 142 Cromwell Tower Barbican, London EC2Y 8DD, UK; 4 Department of Organizational Psychology, Birkbeck, University of London, Malet Street, Bloomsbury, London WC1E 7HX, UK

**Keywords:** Academic Backbone, Secondary school attainment, Undergraduate medical education, Post-graduate medical education, Longitudinal analyses, Continuities, Medical student selection, Cognitive capital, Medical capital, Aptitude tests

## Abstract

**Background:**

Selection of medical students in the UK is still largely based on prior academic achievement, although doubts have been expressed as to whether performance in earlier life is predictive of outcomes later in medical school or post-graduate education. This study analyses data from five longitudinal studies of UK medical students and doctors from the early 1970s until the early 2000s. Two of the studies used the AH5, a group test of general intelligence (that is, intellectual aptitude). Sex and ethnic differences were also analyzed in light of the changing demographics of medical students over the past decades.

**Methods:**

Data from five cohort studies were available: the Westminster Study (began clinical studies from 1975 to 1982), the 1980, 1985, and 1990 cohort studies (entered medical school in 1981, 1986, and 1991), and the University College London Medical School (UCLMS) Cohort Study (entered clinical studies in 2005 and 2006). Different studies had different outcome measures, but most had performance on basic medical sciences and clinical examinations at medical school, performance in Membership of the Royal Colleges of Physicians (MRCP(UK)) examinations, and being on the General Medical Council Specialist Register.

**Results:**

Correlation matrices and path analyses are presented. There were robust correlations across different years at medical school, and medical school performance also predicted MRCP(UK) performance and being on the GMC Specialist Register. A-levels correlated somewhat less with undergraduate and post-graduate performance, but there was restriction of range in entrants. General Certificate of Secondary Education (GCSE)/O-level results also predicted undergraduate and post-graduate outcomes, but less so than did A-level results, but there may be incremental validity for clinical and post-graduate performance. The AH5 had some significant correlations with outcome, but they were inconsistent. Sex and ethnicity also had predictive effects on measures of educational attainment, undergraduate, and post-graduate performance. Women performed better in assessments but were less likely to be on the Specialist Register. Non-white participants generally underperformed in undergraduate and post-graduate assessments, but were equally likely to be on the Specialist Register. There was a suggestion of smaller ethnicity effects in earlier studies.

**Conclusions:**

The existence of the Academic Backbone concept is strongly supported, with attainment at secondary school predicting performance in undergraduate and post-graduate medical assessments, and the effects spanning many years. The Academic Backbone is conceptualized in terms of the development of more sophisticated underlying structures of knowledge ('cognitive capital’ and 'medical capital’). The Academic Backbone provides strong support for using measures of educational attainment, particularly A-levels, in student selection.

## Background

Educational and professional achievements later in life often depend on educational and professional attainments earlier in life. This principle was recognized long ago in the context of education, with a 1924 article in the *Bulletin of the School of Education of Indiana University* saying: 'the best predictor of future achievement is the level of achievement attained at the time of the prediction. The good reader in the elementary school continues to be a good reader throughout junior and high school; the same is true for [a range of other skills]’ [1].

The principle is now often stated as: 'the best predictor of future behaviour is past behaviour’. An early use of the phrase was in the study by Berdie *et al.*[2], who said that: 'the best predictor of future behavior is past behavior. Usually the pupil who has done well in high school will do well in college. Correlations between high school and college grade averages are about .50’.

In the context of medical education, we will refer to this principle as the 'Academic Backbone’. Within medicine and medical science, we believe there is good reason to believe that the post-graduate understanding of, say, respiratory disease, is built upon knowledge, experience, and understanding acquired as a clinical student, which is itself built upon an understanding of pulmonary physiology acquired in the basic medical sciences, which in turn is built upon more basic biological knowledge acquired in Advanced level (A-level) Biology and Chemistry, which has its foundations in science learned at General Certificate of Secondary Education/Ordinary level (GCSE/O-level) and earlier, with those concepts based on earlier educational achievements in the form of being able to read, write, do arithmetic, and so on. In this paper we will assess evidence for the Academic Backbone, looking not only at correlations between secondary school and university grades, but also at correlations of post-graduate performance with secondary school and university grades.

Our metaphorical use of Academic Backbone has two origins. Firstly, just as the human head stands erect, vertical and stably situated above the ground not merely because of the skeletal support provided by the vertebrae, but also because of the dynamic tensions of the muscles and tendons positioned around it, so advanced post-graduate knowledge is developed from and maintained by the interlocking sets of clinical knowledge, practical skills, and theoretical understanding acquired previously during training, not only in the specialist area itself but also in a range of cognate disciplines and skills that together provide the intellectual underpinnings of medical science. Secondly, our use of the term 'backbone’ is not only an anatomical metaphor, but also is inspired by the diagrams often found in structural equation modeling, which we will use later in these analyses, whereby a series of measures are laid side by side, from left to right, each causing the ones to its right, and being caused by the ones to its left, in what can also be envisaged as a backbone around which other factors are located. The statistical correlate of the backbone is what technically is called a simplex of correlations across time, in effect saying that the present is built upon the past, and provides the foundations for the future. Within medical education, the idea of the Academic Backbone is therefore potentially both metaphor and causal reality.

If there is an Academic Backbone, then a key theoretical and practical corollary is that academic attainment should be a major basis for the selection of medical students. It is therefore important to assess both the extent to which measures within undergraduate and post-graduate medical education are predictive of later measures in undergraduate and post-graduate education, and the extent to which selection measures such as results on GCSE, A-level, and intellectual aptitude tests [3] are predictive of undergraduate and post-graduate attainment. Correlations within and between undergraduate and post-graduate attainment measures are relatively straightforward to calculate, even though there are relatively few systematic examples of such measures in the literature. More problematic is the assessment of correlations between measures of attainment prior to medical school entry and measures of undergraduate and post-graduate attainments. Empirically, the collection of the data and the calculation of the correlations is not difficult, but such correlations often seem to be disappointingly small, to the extent that they do not seem to provide a worthwhile basis for selection. This has resulted in statements, found even in prestigious journals, that measures such as A-level grades are actually of little value in predicting university attainment [4]. This would, however, be a naive interpretation. Within psychometrics, the problem of low correlation between performance on a selection test and outcome performance in those selected, is well known. Burt in 1943 [5] referred to the, 'time-honoured fallacy of judging the efficiency of a scholarship examination as a means of *selection* by stating its efficiency as a means of predicting the order of merit *within* the selected group’ (p.2).

The most fundamental problem in studying selection is that those who fail to be selected on the basis of a test are by necessity lower performers on that test, and we can rarely, if ever, measure how they might have done in post-selection tests. In order to validate selection measures such as A-level grades, we need to know about the correlations in the entire pool of applicants, not just in those who have been selected. However, individuals within the pool of applicants who fail to get in to medical school because of poor A-level grades never take medical school examinations, so we can never find out whether, had they been allowed in, they would have done as badly as they did in their A-levels, or whether they would have confounded expectations and done well. This restriction of range means that in the selected group (those who enter medical school) the correlation of the selection measure (for example, A-levels) with the outcome measure (for example, first year medical school examinations) will necessarily be weaker than would be the case if performance were to have been assessed across the whole range of medical school applicants. That situation could only be assessed empirically if entrants were to be a random, representative sample from the pool of all applicants, with A-level grades at all levels of achievement. Statistical solutions to the problem of range restriction have been explored for many decades [5] and the problem is now statistically tractable [6,7], so that validity coefficients for selection as a whole (so-called 'construct validity’) can therefore be calculated.

Although the construct-level predictive validity of tests used in student selection is of fundamental interest, having acknowledged it we will not consider it further here, there being a number of complications in its calculation, and instead we will explore the issue in a separate paper [8], which will build on many of the results described here. Here, we will concentrate on the extent to which the Academic Backbone has empirical substance, manifesting as significant correlations between earlier and later measures of performance, before, within, and after medical school.

Selection measures used in medicine can be broadly divided into measures of attainment (or achievement) and measures of aptitude (or ability) [3]. Attainment/achievement tests, of which GCSEs and A-levels in the UK would be examples, typically assess the knowledge and skills that have been acquired during formal secondary education, and high achievement probably requires not only intellectual ability but also motivation and generic study skills [9]. By contrast, aptitude/ability tests, such as the UK Clinical Aptitude Test (UKCAT) and BioMedical Admissions Test (BMAT) in the UK, emphasize 'intellectual capabilities for thinking and reasoning, particularly logical and analytical reasoning abilities’ [10]. They are felt to be measures of potential and to be independent of formal schooling, and in many ways can be regarded as overlapping with measures of basic mental ability or intelligence. Tests such as the Medical College Admission Test (MCAT), used to select medical students in the USA [11], measure substantive academic understanding of a range of material from biology, chemistry, and physics, and are therefore primarily measures of attainment rather than of aptitude.

Implicit in the use of measures of academic attainment and of aptitude is an assumption that such measures assess skills that underpin and continue to underpin performance both in the undergraduate medical course, and in post-graduate training and professional achievement. The major difference between selection based on aptitude and on attainment measures is that the use of aptitude tests assumes that generic thinking and reasoning skills are the major predictors of medical school performance, whereas the use of attainment tests assumes that substantive knowledge, such as of the facts, theories and ideas of biology or chemistry, are themselves predictors of medical school performance in addition to general skills, and that previous good performance on attainment tests is an indirect indicator of some combination of motivation, intellectual ability, and personality [12].

In the present study, our primary aim was to assess the predictive validity of measures of secondary school attainment in the UK in predicting performance not only in undergraduate medical school examinations, but also in post-graduate training. Because we had access to those data, we particularly considered the Membership of the Royal Colleges of Physicians of the United Kingdom (MRCP(UK)), a major post-graduate medical examination taken by many UK medical graduates, and entry onto the Specialist Register of the UK General Medical Council (GMC). In addition, where possible, we considered data on a standard measure of intellectual ability, the AH5 Group test of General Intelligence, which is specifically aimed at university level students [13], and we compared it with academic attainment.

### Sex and ethnicity as predictors of outcome

Two demographic factors of continuing interest in medical education are sex and ethnicity. Non-white UK medical students perform less well both in medical school examinations [14,15] and in post-graduate examinations [15], including the MRCP(UK) [16]. Men and women also perform differently on the MRCP(UK), men and women being equally likely to pass Part 1, but women being more likely to pass Part 2 and the Practical Assessment of Clinical Examination Skills (PACES) [16]. Men are also far more likely to be investigated and sanctioned by the GMC for Fitness to Practise concerns [17,18]. Interpreting differential performance in post-graduate examinations is complicated by the fact that doctors choose whether or not to take examinations such as MRCP(UK), and those choosing to take an examination may not be a random subset of those graduating from medical school. Post-graduate examinations cannot themselves be used to assess the extent of such processes, because the post-graduate examination boards do not currently have access to information about undergraduate performance. The cohort studies described here did have such background data, and hence differential performance and differential choice could be related to previous performance, sex, and ethnicity. Because we do not wish to detract from the primary emphasis of the current study on the Academic Backbone and predictive validity, we have mainly included analyses of sex and ethnicity in the additional materials, but will discuss their findings in this main paper.

### Overview of the datasets

Our analyses assess the concept of the Academic Backbone in five, separate, longitudinal cohort studies of medical students. Two studies are particularly important, one of which, the 1990 Cohort Study, is very large, the medical students and doctors being followed up on various occasions since 1990. A second study, the University College London Medical School (UCLMS) Cohort Study, whose students entered clinical school in 2005 and 2006, is not as large, but has a more fine-grained follow-up in each year at medical school. Less detailed analyses are also available for three somewhat smaller cohorts, the 1985 cohort, the 1980 cohort, and the Westminster cohort. In each of the datasets, a major interest is the role of GCSE/O-level and A-level results in predicting undergraduate and post-graduate outcomes. A subset of 1990 cohort students was administered an abbreviated version of the AH5 intelligence test [13], and the full version of the AH5 was administered to the students in the Westminster Study. Given the continuing controversy in the UK over the use in selection of aptitude tests for selecting medical students [3,12], the predictive validity of such measures is of some interest.

### Statistical issues

There are several tricky statistical issues in analyzing correlations between attainment measures, and undergraduate and post-graduate outcomes.

### Right-censoring of measures

A growing problem for medical educators in the UK is that there has been grade inflation in both GCSE and A-level examinations, which are taken by the majority of candidates applying to UK medical school. Most applicants take three or more A-levels, which until 2010 were scored as A = 10, B = 8, C = 6, D = 4, E = 2, other = 0. For the best three A-level grades attained, the maximum score is 30, and a growing proportion of students each year are 'at ceiling’ with AAA grades [19]. In statistical terms, A-level grades are 'right-censored’, with the absence of higher grades meaning that many candidates are forced into the top category, even though they would be differentiated with a harder, more stretching and extending assessment. A-level and GCSE grades are therefore skewed to the left and are kurtotic, reducing the standard deviation (SD) and the apparent mean, and also artifactually reducing the size of the correlation with other variables.

### Grouping of measures

Outcome measures in medicine are not always normally distributed and continuous, and sometimes are binary (passed/failed), or ordinal with a small number of categories (honors, pass, or failed/resat). Such grouping of what is implicitly an underlying, normally distributed, latent variable, also means that actual correlations are lower than the true, underlying correlations. Classically, correlations can be calculated as tetrachoric, polychoric, or biserial (not point-biserial) correlations, all of which find the correlation between latent underlying variables.

### The Markov Chain Monte Carlo method for calculating correlations

Given data that are right-censored or based on binary or ordinal measures, no easy analytic solution is available to calculate the underlying correlation, or the means and SDs of the latent variables. A solution is to estimate the parameters using the Markov Chain Monte Carlo (MCMC) algorithm [20], which can not only estimate the latent correlations and the uncensored means and SDs, but also allows estimation of confidence intervals for those parameters. The method also works when measures are binary or ordinal.

## Methods

Five separate longitudinal studies are described and re-analyzed here. Two longitudinal datasets, the UCLMS Cohort Study and the 1990 Cohort Study, were analyzed in detail. In addition, longitudinal data from the 1985 Cohort Study, the 1980 Cohort Study, and the Westminster Cohort Study were also analyzed. Not all measures are available for all studies, but together the datasets provide a picture of selection in UK medical schools over the past three decades.

### The UCLMS Cohort Study

The sampling frame for this study consisted of two groups of entrants to the clinical course (year 3) at UCLMS (then called the Royal Free and University College Medical School; RFUCMS) in September 2005 (n = 383) and 2006 (n=346). Of the total 729 students, 621 (85.2%) had taken their basic medical sciences (BMS) course at UCLMS, with all but one of the remaining 108 students studying BMS at Oxford or Cambridge. Students entering clinical studies in 2005 or 2006 had entered medical school in 2001 (n = 10), 2002 (n = 245), 2003 (n = 352), and 2004 (n = 122), with the different times of entry reflecting personal circumstances, examination failure, or intercalated degrees. Students took Finals in 2007 (n = 270), 2008 (n = 367), 2009 (n = 71), 2010 (n = 6), 2001 (n = 3), or later (in a few cases), with the different dates for taking Finals being due to a range of reasons, including intercalating (clinical) BSc or PhD degrees, examination failure, or personal circumstances. Examination results were collected for all students taking first and second year examinations at UCL, and for all third, fourth, and fifth year examinations. Previous examination results were not available for students entering the third year from Oxford, Cambridge, or elsewhere. A six-page questionnaire asking about a wide range of demographic, social, and psychological variables was distributed at the beginning of the third year as a part of the PhD research for one of the authors (KW) [21], and questionnaires returned by 601 (82.4%) of the 729 students.

### A-levels, GCSEs, and O-levels

The majority of the students in medical schools in England, Wales, and Northern Ireland, as well as some students in Scotland, had taken A-level examinations at the age of 17 years, typically in three subjects but sometimes in four or more. Examinations are graded from A to E (the A* grades not having been introduced at the time of the UCLMS Cohort Study). The conventional scoring method scores A = 10, B = 8, C = 6, D = 4, and E = 2 points, with the three highest grades being summed to give a score with a maximum of 30. In addition, the number of A-levels taken and the mean A-level grade achieved were also analyzed. A-level grades have been gradually climbing over the years (so-called 'grade inflation’), so that grades are much higher for the UCLMS cohort than for the 1990 cohort, with many students in the UCLMS Cohort Study being at the ceiling of 30 points. GCSE examinations are typically taken at the age of 16 years, although they can be taken earlier. GCSE grades at the time of the UCLMS Cohort Study were scored as A* = 6, A = 5, B = 4, C = 3, D = 2, E = 1, and F = 0. GCSEs were scored as mean points per GCSE, total points across all GCSEs taken, and number of GCSEs. Examination results at secondary school (GCSEs and A-levels), as well as basic demographic measures, were obtained from medical school records.

### Medical school performance

Performance of students in each year was summarized by the medical school as a total score, a score on written examination (in the BMS course in years 1 and 2), and a score on practical or objective structured clinical examination (OSCE) (in clinical years 3 to 5). Because students entered the medical school in different years, comparability was ensured by converting all scores to z-scores by year (mean = 0, SD = 1).

### MRCP(UK) results

Performance of the UCLMS Cohort in the MRCP(UK) examinations was obtained from the records of MRCP(UK) Central Office, based on a 'History file’ extracted on October 12, 2012. For the UCLMS Cohort the format of MRCP(UK) consisted of three parts.

The Part 1 examination assesses basic clinical knowledge of medicine along with relevant clinical science, and consists of two 3-hour papers, each containing 100 best-of-five (BOF) assessments. Standard-setting was carried out by Angoff-based criterion-referencing, coupled with a Hofstee compromise method until 2008, when statistical equating using item-response theory (IRT) was introduced.

The Part 2 examination assesses more complex clinical scenarios, often involving detailed biochemical, hematological, or other data, sometimes with ECGs, X-rays, or photographs. The Part 2 examination consists of two three-hour BOF assessments, with Angoff and Hofstee standard-setting as in Part 1 until 2009, after which IRT-based statistical equating was introduced.

The clinical examination, PACES, consists of an OSCE examination with five stations, at each of which the candidates examine real patients, or take histories from or interview simulated patients [22,23]. There are two examiners at each station [22,23], who assess each candidate independently. In 2009, the format of PACES was changed, and the examination was renamed new PACES (nPACES) [24]. The major change was that instead of there being a global assessment at each station, up to seven separate clinical skills were assessed at each station, with candidates having to achieve an overall pass in each of the seven skills.

Despite the various changes in the Part 1, Part 2, and PACES examinations, equivalent marks are straightforwardly available, and can be compared across diets. For Part 1 and Part 2, marks are expressed as percentage points above or below the pass mark (which varies from diet to diet), with multiple true/false (MTF) and BOF questions readily being equated. For PACES, marks are translated into a summed total for the various stations/skills, and expressed as a percentage relative to the pass mark, as described previously [25]. For Part 1, Part 2, and PACES, negative marks indicate a fail, and positive marks a pass. All MRCP(UK) marks were analyzed in relationship to the mark at the first attempt, which other research has shown is a good indicator of overall performance [26].

### The 1990 Cohort Study

The sampling frame for this study consisted of applicants to five different English medical schools in the autumn of 1990. The study surveyed all applicants to five medical schools in England: three in London (St. Mary’s Hospital Medical School, United Medical and Dental Schools of Guy’s and St. Thomas’s (UMDS), and University College and Middlesex School of Medicine (UCMSM)), and two in the north of England (Sheffield and Newcastle-upon-Tyne). The study had information on a total of 6,901 medical school applicants, although not all information was available for all of them. Applicants in 1990 could make up to five applications to medical schools through the Universities Central Council on Admissions (UCCA); now the Universities and Colleges Admissions Service (UCAS), and as a result, the study included applicants and entrants to all of the (then) 28 medical schools in the UK. A total of 3,333 applicants were accepted at a medical school, with 2,962 accepted in 1991, and the majority of the remainder accepted in 1992. The original study included data harvested from UCCA and medical school application forms, and a lengthy questionnaire was also sent to applicants within a week or two of them applying to medical school [27]. The cohort has been followed up since 1990 by questionnaire at four points: when students were in their final year (mostly in 1996 or 1997) [28,29]; in their Pre-Registration House Officer (PRHO) year (mostly in 1997 or 1998) [30,31]; in 2002, when the doctors were mostly working as general practitioners (GPs) or specialist registrars [32];, and again in 2009 [33]. Information about career progression was also obtained from UK medical schools in 1993 to 19944, to ascertain outcome on pre-clinical/BMS courses, and again in 1996 to 1997 to ascertain the outcome in clinical years. GMC numbers for all graduates were identified, and these GMC numbers were subsequently used to link the data with the GMC List of Registered Medical Practitioners (LRMP), and with results from MRCP(UK).

### A-levels, GCSEs, and O-levels

As with the UCLMS Cohort Study, the majority of students in the 1990 cohort took A-level examinations at the age of 17 years. Scoring of A-levels was carried out in the same way as for the UCLMS cohort, with equivalent scores being derived. As already mentioned, there has been considerable grade inflation at A-level over the years, and the mean scores in the 1990 cohort are substantially lower than in the UCLMS cohort, with far fewer students at ceiling. The 1990 Cohort Study took place as GCSEs were being introduced to replace O-levels, and some of the applicants to medical school had GCSEs whereas others had O-levels, and some had both. GCSE examinations for this cohort were scored as A = 5, B = 4, C = 3, D = 2, E = 1, F = 0, the A* grade not being used at the time of the 1990 Cohort Study. As with the UCLMS Cohort, GCSEs were scored as mean points per GCSE, total GCSE points, and number of GCSEs. O-levels were scored in a similar way (A = 5, B = 4, C = 3, D = 2, E = 1, F = 0), although there is no direct comparability with marks awarded for GCSEs. Of the 6,901 applicants in the study, 4,197 had taken only GCSEs, 706 had taken only O-levels, 601 had taken both, and 1,397 had taken neither. Mean points per GCSE or O-level, total points at GCSE/O-level, and number of GCSEs/O-levels taken are therefore only reported for those taking entirely one examination or the other. In order to combine GCSEs and O-levels, mean scores per point on each examination type were converted to z-scores and then treated as a single variable.

### The abbreviated AH5 aptitude test

A subgroup of the applicants who had attended for interview at St. Mary’s, UMDS, or Sheffield took a number of timed psychometric tests, one of which was an abbreviated version of the AH5 test of intelligence, the aAH5 [13]. Having already been selected for interview, it is likely that these applicants were of above-average ability compared with the pool of applicants in general. The aAH5 was entirely for research purposes, and results were not made available to the medical schools concerned.

### Medical school performance

Students in the 1990 Cohort Study were in the 28 different medical schools in the UK, and it was therefore not practical to collect detailed examination data for each student. Instead a simple proforma was sent to the Registrar of each medical school at the end of the pre-clinical/BMS course, and again after Finals, asking for the examination performance of each student in the study to be described on a simple three-point or four-point scale (see Results for more details).

### MRCP(UK) results

MRCP(UK) changed its structure in 2002, after which it was as described above in the section on the UCLMS Cohort Study. Prior to 2002, the Part 1 examination consisted of a 3-hour examination containing 60 five-part MTF questions, with the pass mark determined by norm referencing.

The Part 2 examination had a complex structure. The initial part (Part 2 Written) consisted of a written paper, typically comprising multiple short answers to questions, which were either textual case-histories, included photographs, or had data to be interpreted. Only if the written examination was passed could a candidate go on to the clinical examination (Part 2 Clinical) which contained a long case, a series of short cases, and an oral examination, which were all marked separately, and the results combined. If a sufficient total mark was obtained from the written and clinical examinations, the examination was passed, otherwise both parts had to be taken again.

Most of those in the 1990 cohort took MRCP(UK) before 2002, and so the Part 1 and Part 2 marks are on the old system. Marks on Part 1 are comparable across the old and the new systems, and therefore Part 1 marks apply for whenever the candidate took the examination. Almost no candidates took the post-2002 Part 2 examination or the post-2001 PACES examination as a first attempt at an examination after Part 1, and therefore only pre-2002 results for Part 2 written and clinical are reported here. Marks on MRCP(UK) were identified by linking the database of GMC numbers to an MRCP(UK) database generated in 2009. As with the UCLMS Cohort Study, MRCP(UK) marks were only analyzed for the first attempt at an examination.

### The 1985 Cohort Study

The 1985 Cohort Study [34] used as its sampling frame 2,399 individuals who, in the autumn of 1985, had applied to enter medical school in October 1986, and had included St. Mary’s Hospital Medical School as one of their five university choices. St. Mary’s was a popular choice with applicants, with 24.7% of all medical school applicants including it as one of their five medical school applications, and the study’s 871 entrants included 22.7% of all entrants to UK medical schools in that year. Details of the study have been reported previously, including studies of selection itself [34] and performance in Finals [35]. Both O-level and A-level performance were recorded. Performance on the BMS part of the course was recorded on a four-point scale. For students taking Finals in the (then) constituent schools of the University of London, which had a common, shared examination system, details of performance in all assessments were collected, and aspects of these examinations have been described elsewhere [35]. Results for MRCP(UK) were not available for this cohort, but information was available on whether doctors were on the GMC Specialist Register.

### The 1980 Cohort Study

The 1980 Cohort Study was the first and hence the smallest of the three cohort studies initiated at St. Mary’s Hospital Medical School, with the 1985 and 1990 studies being progressively larger. The sampling frame consisted of the 1,361 individuals who in the autumn of 1980 had applied to study medicine in UK universities, had included St. Mary’s as one of their medical schools, and had a UK correspondence address [36-38]. Applicants included a total of six medical schools on their application form, and overall, 519 students entered a UK medical school, making up 12.9% of all entrants in 1981. BMS performance was recorded on a four-point scale [39]. For students taking the common Finals examinations of the University of London, detailed performance measures were available, as for the 1985 Cohort Study [35].

### The Westminster Cohort Study

The sampling frame for this study consisted of the 511 students entering the clinical course of the Westminster Medical School between 1975 and 1982 [40], and therefore most entered medical school between 1972 and 1980. At that time, the Westminster ran only a clinical course, thus students had carried out their BMS courses elsewhere, and had typically entered medical school 2 or 3 years previously. A-level grades were available for the students. Outcome on the clinical course was recorded on a four-point scale. Follow-up took place in 1989 and again in 2001.

### Statistical analysis

Conventional statistical analyses used IBM SPSS 20 (International Business Machines Corporation, Statistical Package for the Social Sciences, Armonk, New York, USA). Path analyses were conducted to show the Academic Backbone in each cohort. Path coefficients were calculated using multiple regression, with each variable being set in turn as the dependent variable, and all variables to its left as possible causal influences. Paths are included in diagrams when they are significant at *P*<0.05. Path strengths are shown as (standardized) β coefficients from the multiple regression, with the thickness of the arrows being proportional to the path coefficient.

Special-purpose programs were written in MatLab to calculate correlations corrected for right-censoring, as well as tetrachoric and polychoric correlations for grouped data. Examples of the use of the MCMC algorithm for estimating means, SDs, and correlation of bivariately censored data are provided (see Additional file 1: Information file). The programs used the DRAM adaptation of MCMC [41], available from Dr Marko Laine of the University of Helsinki (see helios.fmi.fi/~lainema/mcmc/, helios.fmi.fi/~lainema/mcmc/mcmcstat.zip, and helios.fmi.fi/~lainema/dram/). MCMC analyses typically used a chain length of 5,000, or occasionally 10,000. Parameter estimates were based on the final 2,000 items in the chain, with means and SDs being used as the estimate, and the standard error of parameters, with 5% confidence intervals being estimated as the 2.5th and 97.5th percentiles of the actual values in the chain. Plots of parameter estimates against step number were examined to ensure that ergodic stability had been achieved.

### Ethics

Ethical permission for the studies was provided by the UCL Research Ethics Committee (1980, 1985, 1990, and Westminster Cohorts), and the UCL Committee on the Ethics of Non-NHS Human Research (UCLMS Cohorts). The Chair of the UCL Research Ethics Committee has confirmed that studies such as the present one are generally exempt from needing formal permission from the Committee, being included under section (c) of the exemptions (see http://ethics.grad.ucl.ac.uk/exemptions.php).

## Results

Analysis of the results concentrated on the correlations and path analyses that make up the Academic Backbone. Discussion of sex and ethnic differences will be brief (for more details, see Additional files). Reliability coefficients are often not described or known for the measures reported here, and therefore for each study a section is devoted to estimating reliability. Estimating reliability is good practice for all assessments, and such measures are also needed for estimating construct-level predictive validity, as described elsewhere [8].

### The UCLMS Cohort Study

Of the 729 students, 288 (39.5%) were male and 441 (60.5%) were female. Ethnicity was not known for 14 students, but of the remaining 715, 337 (47.1%) were white and 378 (52.9%) were non-white, using the binary classification used elsewhere [15]. Data on the various examinations in the clinical years (third to fifth) were available for 703 to 723 students, whereas data for BMS (first and second years) were available only for 619 to 621 students (some having taken those examinations elsewhere).

Total GCSE points were available for 599 students, and top three A-level points for 669. Most students (58%) had taken three A-levels, with 62.5% achieving the maximum of 30 points; 16.9%, 12.3%, 3.1%, and 2.2% achieving 28, 26, 24, and 22 points; and 2.9% achieving 20 or fewer points (mean ± SD 28.43 ± 2.77, median 30). The average number of GCSEs taken was 10.04, with students achieving a mean ± SD of 53.6 ± 7.84 points, with an average GCSE grade of 5.32 ± 0.50 points (that is, between and A and an A*). Mean GCSE grade and total GCSE points behave somewhat differently, primarily because mean GCSE points show only a small correlation with number of GCSEs taken (r = 0.096, *P* = 0.018).

Of the original 729 students, 252 (34.6%) had taken MRCP(UK) Part 1 by October 2012, 122 (16.7%) had taken Part 2, and 59 (8.1%) had taken PACES. Part 1, Part 2, and PACES were passed by 80.9%, 90.2%, and 76.3%, respectively, of those taking them. Rates of taking Part 1 were higher for those graduating in 2007 (41.5%; 112/270), compared with 2008 (35.7%; 131/367) and 2009 (12.7%; 9/71). Students attempting MRCP(UK) had significantly higher overall scores in medical school examinations (mean difference: first year: 0.356, *P*<0.001; second year: 0.427, *P*<0.001; third year: 0.388, *P*<0.001; fourth year: 0.425, *P*<0.001; fifth year: 0.520, *P*<0.001), and at A-levels (difference 0.45 points, t_(667)_ = 2.01, *P* = 0.045), but not at GCSE (difference: total GCSE points: 0.503, *P* = 0.456; mean GCSE points: 0.015, *P* = 0.714).

### Correlations between academic measures

Table 1 shows the correlations between the 10 measures constituting the Academic Backbone: GCSEs and A-levels prior to entry into medical school; overall (total) achievement in the 5 years of undergraduate training; and performance in the Part 1, Part 2, and PACES examinations in the MRCP(UK) (for those who had taken it). All correlations were positive, with high correlations between the results in the 5 years of medical school. It is also striking that there were significant correlations of A-levels and even GCSEs with performance in medical school and at MRCP(UK). A-level grades and GCSE grades are strongly right-censored, and Table 1 therefore also shows correlations corrected for right-censorship, when, as expected, the values are much higher than for conventional Pearson correlations.

**Table 1:** **Correlations in the UCLMS Cohort study **^**a,b,c,d**^

	**Mean GCSE grade**^**e**^	**Best three A-levels**^**e**^	**First year**	**Second year**	**Third year**	**Fourth year**	**Fifth year**	**MRCP(UK) Part 1**	**MRCP(UK) Part 2**	**MRCP(UK) PACES**
	**Continuous censored**	**Continuous censored**	**Continuous**	**Continuous**	**Continuous**	**Continuous**	**Continuous**	**Continuous**	**Continuous**	**Continuous**
Mean GCSE grade	1	**0.501**	**0.128**	**0.162**	**0.199**	**0.263**	**0.249**	**0.139**	**0.265**	0.137
		***P*** **<0.001**	***P *** **= 0.003**	***P*** **<0.001**	***P*** **<0.001**	***P*** **<0.001**	***P*** **<0.001**	***P *** **= 0.046**	***P *** **= 0.008**	*P* = 0.331
		**n = 589**	**n = 548**	**n = 547**	**n = 598**	**n = 590**	**n = 583**	**n = 207**	**n = 100**	n = 52
Best three A-levels	**0.561 ± 0.032(0.501 to 0.621)**	1	**0.279**	**0.250**	**0.180**	**0.272**	**0.279**	**0.215**	**0.299**	0.058
			***P*** **<0.001**	***P*** **<0.001**	***P*** **<0.001**	***P*** **<0.001**	***P*** **<0.001**	***P *** **= 0.001**	***P *** **= 0.001**	*P* = 0.676
			**n = 571**	**n = 570**	**n = 668**	**n = 660**	**n = 652**	**n = 232**	**n = 112**	n = 55
First year total	**0.136 ± 0.042(0.054 to 0.214)**	**0.408 ± 0.039(0.337 to 0.482)**	1	**0.752**	**0.502**	**0.522**	**0.550**	**0.559**	**0.497**	0.156
				***P*** **<0.001**	***P*** **<0.001**	***P*** **<0.001**	***P*** **<0.001**	***P*** **<0.001**	***P*** **<0.001**	*P* = 0.307
				**n = 619**	**n = 618**	**n = 608**	**n = 601**	**n = 204**	**n = 94**	n = 45
Second year total	**0.171 ± 0.052** **(0.065 to 0.252)**	**0.328 ± 0.039** **(0.250 to 0.408)**	*	1	**0.523**	**0.590**	**0.583**	**0.595**	**0.501**	0.273
					***P*** **<0.001**	***P*** **<0.001**	***P*** **<0.001**	***P*** **<0.001**	***P*** **<0.001**	*P* = 0.070
					**n = 618**	**n = 608**	**n = 601**	**n = 204**	**n = 94**	n = 45
Third year total	**0.213 ± 0.046** **(0.118 to 0.294)**	**0.265 ± 0.038** **(0.186 to 0.337)**	*	*	1	**0.733**	**0.690**	**0.522**	**0.469**	**0.461**
						***P*** **<0.001**	***P*** **<0.001**	***P*** **<0.001**	***P*** **<0.001**	***P*** **<0.001**
						**n = 608**	**n = 703**	**n = 252**	**n = 122**	**n = 59**
Fourth year total	**0.285 ± 0.039** **(0.202 to 0.362)**	**0.350 ± 0.038** **(0.276 to 0.418)**	*	*	*	1	**0.831**	**0.665**	**0.660**	**0.535**
							***P*** **<0.001**	***P*** **<0.001**	***P*** **<0.001**	***P*** **<0.001**
							**n = 703**	**n = 252**	**n = 122**	**n = 59**
Fifth year total	**0.265 ± 0.038** **(0.194 to 0.349)**	**0.357 ± 0.033** **(0.292 to 0.415)**	*	*	*	*	1	**0.715**	**0.673**	**0.484**
								***P*** **<0.001**	***P*** **<0.001**	***P*** **<0.001**
								**n = 251**	**n = 122**	**n = 59**
MRCP(UK) Part 1	**0.23 9 ± 0.085** **(0.063 to 0.397)**	**0.346 ± 0.088** **(0.177 to 0.514)**	*	*	*	*	*	1	**0.775**	**0.429**
									***P*** **<0.001**	***P *** **= 0.001**
									**n = 122**	**n = 59**
MRCP(UK) Part 2	**0.358 ± 0.123** **(0.094 to 0.592)**	**0.548 ± 0.111** **(0.297 to 0.741)**	*	*	*	*	*	*	1	**0.410**
										***P *** **= 0.001**
										**n = 58**
MRCP(UK) PACES	0.005 ± 0.177 (-0.335 to 0.399)	0.140 ± 0.186 (0.221 to 0.495)	*	*	*	*	*	*	*	1

*Abbreviations*: A-level, Advanced level; GCSE, General Certificate of Secondary Education; MCMC, Markov Chain Monte Carlo; MRCP(UK), Membership of the Royal College of Physicians of the United Kingdom; PACES, Practical Assessment of Clinical Examination Skills; UCLMS, University College London Medical School.

^a^Correlations in entrants to medical school between measures of academic and professional attainment in the UCLMS Cohort Study.

^b^Correlations above the diagonal are simple Pearson correlations and have different values of n for various reasons.

^c^Correlations shown in bold are significant at *P*<0.05.

^d^Correlations in the lower triangle involving censored variables were corrected for censoring using an MCMC method, with 95% confidence intervals calculated from the final 2000 MCMC steps in a chain of 5000.

^e^Values in these two columns are mean ± SE (95% CI) unless otherwise stated.

*Indicates correlations below the diagonal, which are based on two non-censored, continuous variables and therefore identical to those above the diagonal.

### Reliability of medical school examinations

Cronbach’s α, calculated for a composite of the five total marks for the five medical school years was 0.890, indicating good reliability. A similar calculation for a composite of the five written marks from each year gave 0.909, and a composite of the five OSCE/practical marks from the 5 years had 0.796. Likewise, a composite of the four BMS examinations had a reliability of 0.904 (based on two written and two OSCE marks), and a composite of the six clinical examinations had a reliability of 0.913 (based on three written and three OSCE marks). Estimates of the reliabilities of individual assessments could be back-calculated from the Spearman-Brown formula and were found to be 0.618 for a single year total (0.666 for a single written examination, and 0.438 for a single OSCE/practical examination).

Such estimates to some extent are conservative, as they confound within-examination reliability with between-year reliability, and the latter may be less for various reasons. In a recent set of UCL Finals, there were two written (multiple choice question (MCQ)) assessments which had KR20 reliabilities of 0.762 and 0.746, giving an overall reliability of about 0.86, somewhat higher than the estimate here. No reliability was available for the OSCE assessments.

### Written and OSCE assessments

Marks on written examinations were available for all five medical school years, and in all years there were also practical/OSCE assessments, which were practical examinations in years 1 and 2, and clinical examinations in years 3, 4 and 5. A factor analysis of the five marks from written examinations and the five marks from OSCE/practical examinations found a very steep scree-slope (eigenvalues of 5.99, 1.37, 0.68, 0.47, 0.39, 0.33, 0.26, 0.20, 0.17, and 0.14) with a large first factor and a hint of a second factor. Extraction of two factors found no evidence that the second factor was related to a difference between written and OSCE assessments, but instead related to a difference between years 1 and 2 (BMS), and years 3, 4, and 5 (clinical). Scores were calculated for overall performance at medical school, and for performance on each of the examination types (BMS, clinical, written and OSCE/practical) (Table 2).

**Table 2:** **Correlation in the UCLMS Cohort study**^**a,b,c**^

	**Overall performance at medical school (years 1 to 5)**	**Examination performance**			
		**BMS (years 1 and 2)**	**Clinical (years 3 to 5)**	**Written (years 1 to 5)**	**OSCE/practical (years 1 to 5)**
Number of GCSEs	-0.043	-0.034	-0.045	-0.055	-0.027
	*P* = 0.288	*P* = 0.422	*P* = 0.269	*P* = 0.177	*P* = 0.501
	n = 605	n = 552	n = 604	n = 605	n = 605
Mean points per GCSE	**0.262**	**0.156**	**0.260**	**0.263**	**0.247**
	***P*** **<0.001**	***P*** **<0.001**	***P*** **<0.001**	***P*** **<0.001**	***P*** **<0.001**
	**n = 599**	**n = 548**	**n = 598**	**n = 599**	**n = 599**
Mean points per GCSE (corrected for censoring)^d^	**0.277 ± 0.040 (0.204 to 0.360)**	**0.165 ± 0.0410 (0.092 to 0.250)**	**0.267 ± 0.035 (0.204 to 0.342)**	**0.274 ± 0.038 (0.202 to 0.356)**	**0.264 ± 0.039 (0.189 to 0.333)**
Total GCSE points	**0.128**	**0.083**	**0.117**	**0.120**	**0.130**
	***P *** **= 0.002**	***P *** **= 0.053**	***P *** **= 0.004**	***P *** **= 0.003**	***P *** **= 0.002**
	**n = 597**	**n = 546**	**n = 596**	**n = 597**	**n = 597**
Number of A-levels	**0.137**	**0.154**	**0.106**	**0.148**	**0.112**
	***P*** **<0.001**	***P*** **<0.001**	***P*** **<0.004**	***P*** **<0.001**	***P *** **= 0.004**
	**n = 667**	**n = 570**	**n = 667**	**n = 667**	**n = 667**
Mean A-level grade	**0.311**	**0.286**	**0.266**	**0.339**	**0.279**
	***P*** **<0.001**	***P*** **<0.001**	***P*** **<0.001**	***P*** **<0.001**	***P*** **<0.001**
	**n = 668**	**n = 570**	**n = 667**	**n = 667**	**n = 667**
Total points for three best A-levels	**0.301**	**0.282**	**0.257**	**0.331**	**0.272**
	***P*** **<0.001**	***P*** **<0.001**	***P*** **<0.001**	***P*** **<0.001**	***P*** **<0.001**
	**n = 668**	**n = 571**	**n = 668**	**n = 668**	**n = 668**
Total points for three best A-levels (corrected for censoring)^d^	**0.412 ± 0.045 (0.318 to 0.506)**	**0.396 ± 0.041 (0.309 to 0.473)**	**0.342 ± 0.036 (0.260 to 0.404)**	**0.444 ± 0.039(0.368 to 0.511)**	**0.362 ± 0.036 (0.281 to 0.443)**
MRCP(UK) Part 1 mark	**0.709**	**0.610**	**0.685**	**0.733**	**0.646**
	***P*** **<0.001**	***P*** **<0.001**	***P*** **<0.001**	***P*** **<0.001**	***P*** **<0.001**
	**n = 252**	**n = 204**	**n = 252**	**n = 252**	**n = 252**
MRCP(UK) Part 2 mark	**0.656**	**0.526**	**0.651**	**0.669**	**0.625**
	***P*** **<0.001**	***P*** **<0.001**	***P*** **<0.001**	***P*** **<0.001**	***P*** **<0.001**
	**n = 122**	**n = 94**	**n = 252**	**n = 252**	**n = 252**
MRCP(UK) PACES mark	**0.448**	0.220	**0.525**	**0.399**	**0.442**
	***P*** **<0.001**	*P* = 0.146	***P*** **<0.001**	***P *** **= 0.002**	***P*** **<0.001**
	**n = 59**	n = 45	**n = 59**	**n = 59**	**n = 59**

*Abbreviations*: A-level, Advanced level; GCSE, General Certificate of Secondary Education; MRCP(UK), Membership of the Royal College of Physicians of the United Kingdom; OSCE, objective structured clinical examination; PACES, Practical Assessment of Clinical Examination Skills; UCLMS, University College London Medical School.

^a^Pearson correlations of measures of performance at medical school with GCSEs, A-levels, and MRCP(UK).

^b^For n = 600, the standard error of a correlation of 0.1, 0.2, 0.3, 0.4, 0.5, 0.6, and 0.7 is about 0.040, 0.039, 0.037, 0.034, 0.031, 0.026, and 0.021, respectively.

^c^Values significant at *P*<0.05 are shown in bold.

^d^Correlations corrected for right-censorship are shown as the mean estimate ± standard error (95% CI).

The written and OSCE examinations correlated 0.849 (*P*<0.001, n = 726). Given the reliabilities of the written and OSCE examinations (see above), the disattenuated correlation between written and OSCE examinations was 0.849/√(0.909 × 0.796) = 0.998. Written and OSCE examinations can therefore be construed as largely assessing identical constructs, and that is supported by the similarity of their correlations with GCSE, A-level, and MRCP(UK) performance (Table 2), although there is a suggestion that written examination results predict Part 1 better and OSCE results predict PACES better. For completeness, Table 2 also shows correlations with mean GCSE grade and best three A-level grades, corrected for the effect of right-censorship.

BMS and clinical examinations also correlated 0.636 (*P*<0.001, n = 618). Correction for attenuation due to unreliability gave a disattenuated correlation of 0.636/√(0.904 × 0.913) = 0.700, suggesting that BMS and clinical examination performance are separate constructs. Correlations of BMS and clinical marks suggest that GCSEs are better at predicting clinical performance than they are at predicting BMS performance; multiple regression of clinical performance on mean GCSE points and points at best three A-levels gave β coefficients of 0.204 (*P*<0.001) and 0.119 (*P* = 0.010) respectively, whereas BMS performance was only predicted by three best A-levels (β = 0.283, *P*<0.001), and GCSEs were not significant (β = 0.020, *P* = 0.668). BMS and clinical performance both predicted MRCP(UK) Part 1 (β = 0.293 and 0.468, respectively; both *P*<0.001), whereas Part 2 was mostly predicted by clinical performance (β = 0.485, *P*<0.001) and hardly at all by BMS (β = 0.214, *P* = 0.044), and PACES performance was only predicted by clinical performance (β = 0.589, *P*<0.001), and not at all by BMS (β= -0.138, *P* = 0.411).

### The Academic Backbone

**Figure 1:**
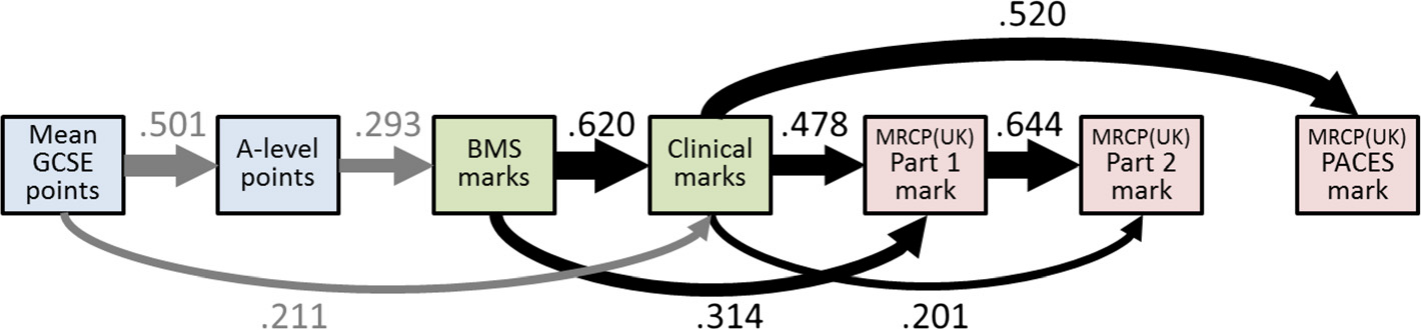
**The Academic Backbone in the UCLMS Cohort Study.** This figure, and Figures 2, 3, 4, and 5, which show path analyses of the Academic Backbone in the various cohorts, have the same structures and conventions, and are also very similar to the figures in the additional material (see Additional files). Pale blue boxes indicate measures obtained prior to medical school, usually at secondary school, pale green boxes indicate performance at medical school, and pale purple boxes indicate post-graduate performance. The path model was fitted using multiple regression, each variable being regressed on all variables to its left (that is, causally prior), using backwards regression, variables being eliminated sequentially until all remaining variables were significant with *P*<0.05. Path coefficients are shown as β coefficients (that is, they are standardized), and arrow thickness is proportional to effect size. Solid black arrows indicate positive β coefficients. Solid arrows entering or leaving secondary school measures are in grey to indicate that they are not accurate estimates of the true effect in the non-selected population. No paths were found which were significant and had negative β coefficients. When interpreting path models, it should be remembered that any analysis towards the right of the diagram takes account of prior effects occurring to the left of the diagram. For this figure, that means, for instance, that the effect of BMS marks on MRCP(UK) Part 1 mark takes into account and is additional to the effect of clinical marks on MRCP(UK) Part 1 mark. Abbreviations: A-level, Advanced level; GCSE, General Certificate of Secondary Education; MRCP(UK), Membership of the Royal College of Physicians of the United Kingdom; PACES, Practical Assessment of Clinical Examination Skills; UCLMS, University College London Medical School.

Figure 1 shows a path diagram indicating the Academic Backbone for the UCLMS cohorts study. The boxes indicate performance in GCSE and A-level examinations, at BMS and clinical examinations at medical school, and in Parts 1, 2, and PACES of MRCP(UK). Path coefficients were calculated as described in statistical methods. With the sole exception of the path from Part 2 to PACES (where n is relatively small), all paths from one variable to the next are significant and large. In addition, there are some effects that have longer-lasting effects, with GCSE points predicting clinical marks, over and above their effect on A-level points; BMS marks influencing performance in Part 1 (over and above their effect via clinical marks); and clinical marks influencing both Part 2 and PACES, over and above their effect via Part 1. Future performance is therefore dependent to a large extent on previous performance. It should be noted that the influences of GCSE points on A-levels, and of A-levels upon BMS marks (and so on) are only estimates calculated for the students who entered medical school. Because GCSEs and A-levels are used in selecting which students should enter medical school, the predictive validity in the pool of all medical school applicants is of greater interest, and this will be higher than the values shown in Figure 1, which inevitably have restriction of range. Calculations of the correlations in the unrestricted population are presented below.

### Sex and ethnicity effects

Of 715 UCLMS medical students, 60.6% were female, a proportion that was not significantly different in white (209/337; 62.0%) and non-white students (224/378; 59.3%; χ^2^ = 0.568, degrees of freedom (df) = 1, *P* = 0.451). Information on student performance broken down by sex and ethnicity, and the path diagram, are provided in detail (see Additional file 2: Information). Males underperformed somewhat at GCSE, and taking that into account, did slightly better at A-level. Males performed better at BMS examinations, but then performed less well on clinical assessments, while once again performing better at MRCP(UK) Part 1. Non-white participants had slightly higher A-level grades, although that was not significant after taking sex into account. Non-white students underperformed on both BMS and clinical assessments, but had equivalent performance on MRCP(UK).

### The 1990 Cohort Study

The 1990 Cohort Study is more complex than the UCLMS Cohort Study, because it is a study of selection, rather than being only a study of entrants. Some measures can therefore be considered either in the overall pool of applicants, or in the restricted group of entrants.

Of the 6,901 medical school applicants in the 1990 Cohort Study, 3,428 (49.7%) were male. Ethnicity was known for 5,341 applicants, of whom 3,614 (67.7%) were white, and 1727(32.3%) were non-white. White applicants were more likely to be female (1955/3614; 54.1%) than were non-white applicants (790/1727; 45.7%); χ^2^ = 32.62, df = 1, *P*<0.001). Of the 3,333 medical school entrants, 1,683 (50.5%) were female. Of the 2,985 entrants whose ethnicity was known, 754 (25.3%) were non-white, female entrants being more common among white entrants (1187/2231; 53.2%) than non-white entrants (333/754; 44.2%; χ^2^ = 18.4, df = 1, *P*<0.001). GCSE or O-level results were available for 4,903 applicants and 2,730 entrants, and A-level results were available for 6,059 applicants and 3,199 entrants. BMS/pre-clinical outcome measures were available for 3,223 entrants, of whom 177 were known to have left the medical school or been asked to leave because of examination failure. A Finals outcome measure was available for 2,509 entrants. GMC numbers were known for 2,823 participants, and 1,077 participants are known to have taken MRCP(UK) on at least one occasion. In December 2012, 1,308 participants were known to be on the GMC Specialist Register, 1094 on the GMC GP Register, with 8 on both registers.

A-level results were coded as three best A-level grades, and were available for 6,059 applicants and 3,193 entrants. Most applicants (59.5%) had taken three A-levels, with 14.1% taking four, 13.0% taking five or more, and 13.4% taking either none (11.3%) or only one or two (2.1%). Of applicants with three or more A-levels, the number of points (mean ± SD) was 20.0 ± SD 8.2 (median 22), with 12.4% gaining the maximum score of 30 points. Of the 3,199 entrants with three or more A-levels, the score for the best three A-levels (mean ± SD) was 24.8 ± 4.92 points (median 26), with 21.3% of entrants gaining the maximum score of 30 points.

Of the total number of applicants, 706 had taken O-levels only, 4,197 had taken GCSEs only, and 601 had taken a mixture of O-levels and GCSEs. For the present purposes, we included only applicants who had taken O-levels only or GCSEs only. The mean grade at O-level or GCSE was then converted to a z-score, and subsequent analyses used the z-scores, irrespective of whether they were from O-levels or GCSEs. It is not possible to compare GCSE grades directly with current GCSE grades because A* grades were not available when the 1990 cohort took GCSEs.

### Reliabilities

No reliability measures were available for either the BMS results or the Finals results.

### Correlations between academic measures

Table 3 shows the correlations in entrants to medical school between the nine measures constituting the Academic Backbone: O-levels/GCSEs, A-levels, and aAH5 prior to entry into medical school; a summary of performance in BMS and clinical examinations in medical school; performance on Part 1, Part 2 Written, and Part 2 Clinical in the MRCP(UK) (for those who had taken it); and being on the Specialist Register. All correlations were positive, and most were significant, the exceptions being for the aAH5 test of aptitude. Once again it is striking that there were significant correlations of A-levels and even O-levels/GCSEs with performance in medical school and at MRCP(UK), and being on the Specialist Register. A-level and O-level/GCSE grades are right-censored, and several of the outcome measures had only small numbers of categories and are ordinal. The correlations below the diagonal in Table 3 were therefore corrected both for censorship and are tetrachoric/polychoric/biserial correlations as appropriate. As expected, the values are somewhat higher than for conventional Pearson correlations.

**Table 3:** **Correlations in the 1990 Cohort Study**^**a,b,c,d,e**^

	**aAH5**	**Mean GCSE/O-level grade**	**Best three A-levels**	**BMS outcome**	**Finals outcome**	**MRCP(UK) Part 1**	**MRCP(UK) Pt 2 Written**	**MRCP(UK) Pt 2 Clinical**	**On Specialist Register**
	**Continuous**	**Continuous censored**	**Continuous censored**	**Ordinal**	**Ordinal**	**Continuous**	**Continuous**	**Continuous**	**Binary**
aAH5	1	**0.203**	**0.179**	0.037	0.051	0.126	**0.276**	**0.179**	0.037
		***P*** **<0.001**	***P*** **<0.001**	*P* = 0.313	*P* = 0.216	*P* = 0.051	***P*** **<0.001**	***P *** **= 0.025**	*P* = 0.339
		**n = 714**	**n = 762**	n = 766	n = 597	n = 242	**n = 193**	**n = 156**	n = 670
Mean GCSE/O-level grade	**0.223 ± 0.034 (0.155 to 0.292)**	1	**0.250**	**0.141**	**0.138**	**0.245**	**0.096**	**0.235**	**0.047**
			***P*** **<0.001**	***P*** **<0.001**	***P*** **<0.001**	***P*** **<001**	***P *** **= 0.014**	***P*** **<0.001**	***P *** **= 0.022**
			**n = 2662**	**n = 2657**	**n = 2072**	**n = 829**	**n = 660**	**n = 526**	**n = 2351**
Best three A-levels	**0.190 ± 0.036 (0.117 to 0.264)**	**0.561 ± 0.007(0.548 to 0.573)**	1	**0.128**	**0.100**	**0.230**	**0.085**	**0.096**	**0.147**
				***P*** **<0.001**	***P*** **<0.001**	***P*** **<0.001**	***P *** **= 0.019**	***P *** **= 0.019**	***P*** **<0.001**
				**n = 3096**	**n = 2413**	**n = 957**	**n = 753**	**n = 597**	**n = 2664**
BMS outcome	**0.050 ± 0.042 (-0.033 to 0.131)**	**0.192 ± 0.206 (0.134 to 0.237)**	**0.191 ± 0.018 (0.151 to 0.227)**	1	**0.210**	**0.296**	**0.111**	**0.102**	**0.097**
					***P*** **<0.001**	***P*** **<0.001**	***P *** **= 0.002**	***P *** **= 0.012**	***P*** **<0.001**
					**n = 2506**	**n = 989**	**n = 777**	**n = 614**	**n = 2760**
Finals outcome	**0.055 ± 0.058 (-0.066 to 0.155)**	**0.202 ± 0.023 (0.154 to 0.241)**	**0.167 ± 0.025 (0.115 to 0.219)**	**0.391 ± 0.033 (0.322 to 0.453)**	1	**0.281**	**0.167**	**0.188**	**0.143**
						***P*** **<0.001**	***P*** **<0.001**	***P*** **<0.001**	***P*** **<0.001**
						**n = 872**	**n = 698**	**n = 571**	**n = 2337**
MRCP(UK) Part 1	**0.120 ± 0.062 (0.002 to 0.249)**	**0.272 ± 0.032 (0.209 to 0.331)**	**0.256 ± 0.032 (0.184 to 0.314)**	**0.384 ± 0.034 (0.321 to 0.452)**	**0.352 ± 0.039 (0.269 to 0.427)**	1	**0.208**	**0.182**	**0.253**
							***P*** **<0.001**	***P*** **<0.001**	***P*** **<0.001**
							**n = 730**	**n = 571**	**n = 938**
MRCP(UK) Part 2 written (old format)	**0.270 ± 0.067 (0.140 to 0.393)**	**0.115 ± 0.035 (0.049 to 0.184)**	**0.067 ± 0.039 (-0.020 to 0.138)**	**0.170 ± 0.055 (0.061 to 0.278)**	**0.214 ± 0.045 (0.134 to 0.305)**	**0.202 ± 0.034 (0.133 to 0.271)**	1	**0.233**	**0.086**
								***P*** **<0.001**	***P *** **= 0.019**
								**n = 606**	**n = 743**
MRCP(UK) Part 2 clinical (old format)	**0.189 ± 0.072 (0.034 to 0.321)**	**0.249 ± 0.047 (0.158 to 0.339)**	**0.119 ± 0.047 (0.031 to 0.208)**	**0.164 ± 0.063 (0.051 to 0.286)**	**0.233 ± 0.048 (0.126 to 0.326)**	**0.175 ± 0.039 (0.091 to 0.247)**	**0.245 ± 0.040 (0.166 to 0.321)**	1	**0.120**
									***P*** **<0.004**
									**n = 582**
On Specialist Register	**0.032 ± 0.047 (-0.064 to 0.120)**	**0.025 ± 0.017 (-0.018 to 0.058)**	**0.200 ± 0.027 (0.143 to 0.243)**	**0.160 ± 0.032 (0.099 to 0.217)**	**0.240 ± 0.033 (0.176 to (0.303)**	**0.317 ± 0.03 7(0.250 to 0.391)**	**0.116 ± 0.048 (0.0228 to 0.209)**	**0.163 ± 0.054 (0.051 to 0.273)**	1

*Abbreviations*: aAH5, Abbreviated AH5; A-level, Advanced level; BMS, Basic medical sciences; GCSE, General Certificate of Secondary Education; MCMC, Markov Chain Monte Carlo; MRCP(UK), Membership of the Royal College of Physicians of the United Kingdom.

^a^Correlations in entrants to medical school between measures of academic and professional attainment attainment in the 1990 Cohort study.

^b^Correlations above the diagonal are simple Pearson correlations (for example, point-biserials, φ correlations, where a measure is binary or ordinal), and have different n values for various reasons.

^c^Correlations shown in bold are significant at *P*<0.05.

^d^Correlations in the lower triangle were calculated taking account of censoring, and for ordinal values are equivalent to tetrachoric/biserial/polychoric correlations.

^e^Values are mean ± SD (95% CI) unless otherwise stated, and the 95% CIs are the result of the final 2000 MCMC steps in a chain of 5000.

### The Academic Backbone

**Figure 2:**
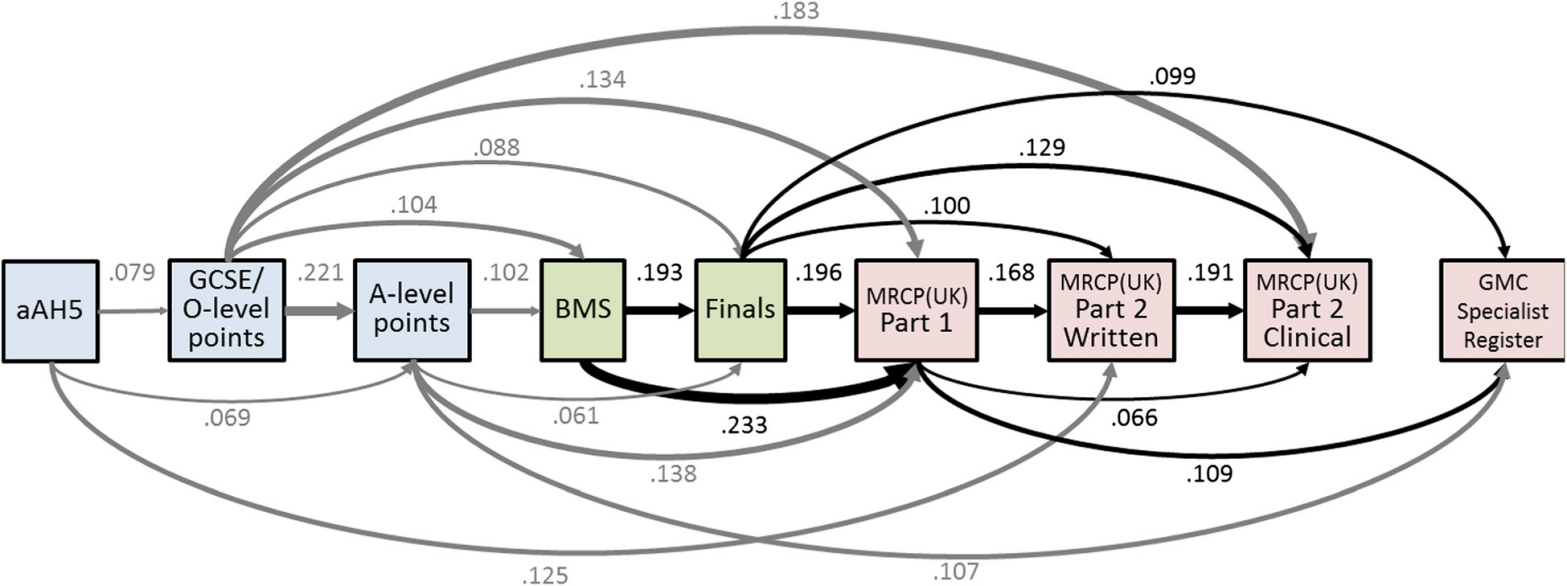
**The Academic Backbone in the 1990 Cohort Study.** This figure, and Figures 1, 2, 3, 4, and 5, which show path analyses of the Academic Backbone in the various cohorts, have the same structures and conventions, and are also very similar to the figures in the additional material (see Additional files). Pale blue boxes indicate measures obtained prior to medical school, usually at secondary school, pale green boxes indicate performance at medical school, and pale purple boxes indicate post-graduate performance. The path model was fitted using multiple regression, each variable being regressed on all variables to its left (that is, causally prior), using backwards regression, variables being eliminated sequentially until all remaining variables were significant with *P*<0.05. Path coefficients are shown as β coefficients (that is, they are standardized), and arrow thickness is proportional to effect size. Solid black arrows indicate positive β coefficients. Solid arrows entering or leaving secondary school measures are in grey to indicate that they are not accurate estimates of the true effect in the non-selected population. No paths were found which were significant and had negative β coefficients. When interpreting path models, it should be remembered that any analysis towards the right of the diagram takes account of prior effects occurring to the left of the diagram. For this figure, that means, for instance, that the effect of BMS marks on MRCP(UK) Part 1 mark takes into account and is additional to the effect of clinical marks (Finals) on MRCP(UK) Part 1 mark. Abbreviations: A-level, Advanced level; GCSE, General Certificate of Secondary Education; MRCP(UK), Membership of the Royal College of Physicians of the United Kingdom; PACES, Practical Assessment of Clinical Examination Skills; UCLMS, University College London Medical School.

Figure 2 shows the Academic Backbone path diagram for the 1990 Cohort Study. As with Figure 1, the boxes indicate performance in O-levels/GCSE and A-level examinations, in BMS and clinical examinations at medical school, and in Part 1, Part 2, and PACES of MRCP(UK); the aAH5 and the Specialist Register are also included. All direct paths from aAH5 to MRCP(UK) Part 2 clinical are significant, and in addition, many indirect paths are also significant. A particularly notable point is that O-levels/GCSEs are predictive of performance both at undergraduate and post-graduate level, with the effect being significant after A-levels are taken into account. Once again, future performance is dependent to a large extent on previous performance. It should also be remembered that the influences of GCSE points on A-levels, and of A-levels upon BMS marks (and so on), are only estimates calculated for the students who entered medical school, and these effects are therefore indicated in figure in gray. Calculations of the correlations in the unrestricted population will be presented below.

### Sex and ethnicity effects

Sex and ethnicity influence performance at several different stages of undergraduate and post-graduate performance, and these are described in detail (see Additional file 2: Information). Because of the large sample size, many effects are significant. As with the UCLMS cohort, male participants had lower GCSE scores and somewhat higher A-level scores, underperformed at BMS and clinical assessments, were less likely to attempt MRCP(UK), and performed less well at the MRCP(UK) clinical examination, but were more likely to be on the Specialist Register. Non-white participants performed less well on both GCSEs and the aAH5, but somewhat better at A-levels after taking GCSEs and aAH5 into account. They then underperformed in BMS and Finals assessments, and in all three parts of MRCP(UK), but were equally as likely as whites to be on the Specialist Register.

### The 1985 Cohort Study

Like the 1990 Cohort Study, the 1985 Cohort Study is a study of selection, and therefore data are available for both applicants and entrants. Of 2,399 individuals in the original sampling frame, 55.3% were male and 44.7% female. Ethnicity was known in 2032 cases, with 71.8% being white and 28.2% non-white. The number of applicants accepted at a UK medical school was 919 (38.3%), with 45.2% being female and 17.1% (146/854) from ethnic minorities. Outcome at the end of the BMS course was known in 880 cases (with a further 15 being exempt from BMS examinations): 103 (11.7%) gained a distinction (score 4), 469 (53.3%) were described as 'satisfactory’ (score 3), 249 (28.3%) had to resit one or more examinations (score 2), and 59 (6.7%) failed and had to leave the medical school (score 1). Results for finals were available for only 361 students who took their examinations in the University of London, with separate scores being available for overall performance (first principle component of all the individual measures), and separate scores for different types of examination (MCQ, clinical examinations, and oral examinations) and for different subjects (Medicine, Surgery, Pathology, Pharmacology, and Obstetrics and Gynecology). There were 813 students who were known to have qualified because at some time they had GMC registration numbers, but 67 were known by 2009 to have dropped off the Register for various reasons, including death, emigration, and suspension. In 2009, of 760 doctors on the Register, 277 (64.4%) were on the GP Register, 421 (55.4%) were on the Specialist Register, and 62 were on neither Register.

A-level results were available for 2005 applicants, with the mean ± SD for three A-levels being 19.9 ± 7.06 points (median 20), and 184 (9.2%) having the maximum of 30 points. Of 884 entrants with A-levels, the mean number of points was 25.3 ± 3.95 (median 26), with 165 (18.7%) having the maximum of 30 points. Of the applicants, 2020 had six or more O-level results, with the mean grade being 4.00 ± 0.61 (5 was the maximum possible score; grade A).

### Reliabilities

No reliability measure is available for the BMS examinations. The total mark for the Finals examination, which comprised 25 separate standardized individual marks from various papers, gave a Cronbach’s α of 0.897 based on 358 candidates.

### Correlations between academic measures

Table 4 shows the correlations for entrants to medical school between the five measures constituting the Academic Backbone: O-levels, A-levels, performance in medical school BMS and clinical examinations, and being on the Specialist Register. All of the correlations were positive, and most were significant. As before, A-levels and also O-levels correlated with performance in medical school, which in turn correlated with being on the Specialist Register. Correlations in the lower triangle show values corrected for right-censorship and for categorical variables.

**Table 4:** **Correlations in the 1985 Cohort Study**^**a,b,c, d,e**^

	**Mean GCSE/O-level grade**	**Best three A-levels**	**BMS outcome**	**Finals outcome**	**On Specialist Register**
	**Continuous censored**	**Continuous censored**	**Ordinal**	**Continuous**	**Binary**
Mean GCSE/O-level grade	1	**0.381**	**0.214**	**0.222**	0.067
		***P*** **<0.001**	***P*** **<0.001**	***P*** **<0.001**	*P* = 0.071
		**n = 848**	**n = 843**	**n = 348**	n = 731
Best three A-levels	**0.390 ± 0.031 (0.327 to 0.446)**	1	**0.180**	**0.240**	0.066
			***P*** **<0.001**	***P*** **<0.001**	*P* = 0.075
			**n = 848**	**n = 347**	n = 733
BMS outcome	**0.242 ± 0.033 (0.184 to 0.314)**	**0.216 ± 0.037 (0.147 to 0.290)**	1	**0.350**	**0.096**
				***P*** **<0.001**	***P *** **= 0.009**
				**n = 361**	**n = 742**
Finals outcome (London only)	**0.234 ± 0.046 (0.140 to 0.323)**	**0.254 ± 0.055) (0.148 to 0.362)**	**0.411 ± 0.054 (0.282 to 0.508)**	1	**0.214**
					***P*** **<0.001**
					**n = 334**
On Specialist Register	**0.088 ± 0.045 (0.003 to 0.174)**	**0.089 ± 0.042 (0.012 to 0.175)**	**0.129 ± 0.051 (0.030 to 0.226)**	**0.267 ± 0.062 (0.136 to 0.385)**	1

*Abbreviations*: A-level, Advanced level; BMS, Basic medical sciences; GCSE, General Certificate of Secondary Education; MCMC, Markov Chain Monte Carlo; O-level, Ordinary level.

^a^Correlations in entrants to medical school between measures of academic and professional attainment attainment in the 1985 Cohort study.

^b^Correlations above the diagonal are simple Pearson correlations (for example, point-biserials, φ correlations. where a measure is binary or ordinal), and have different n values for various reasons.

^c^Correlations shown in bold are significant at *P*<0.05.

^d^Correlations in the lower triangle were calculated taking account of censoring, and for ordinal values are equivalent to tetrachoric/polychoric correlations.

^e^Values are mean ± SD (95% CI) unless otherwise stated, and the 95% confidence intervals are the result of the final 2000 MCMC steps in a chain of 5000.

### The academic backbone

**Figure 3:**
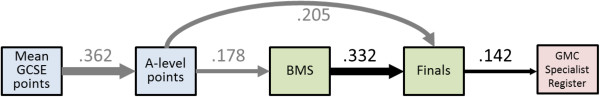
**The Academic Backbone in the 1985 Cohort Study.** This figure, and Figures 1,2, 4, and 5, which show path analyses of the Academic Backbone in the various cohorts, have the same structures and conventions, and are also very similar to the figures in the additional material (see Additional files). Pale blue boxes indicate measures obtained prior to medical school, usually at secondary school, pale green boxes indicate performance at medical school, and pale purple boxes indicate post-graduate performance. The path model was fitted using multiple regression, each variable being regressed on all variables to its left (that is, causally prior), using backwards regression, variables being eliminated sequentially until all remaining variables were significant with *P*<0.05. Path coefficients are shown as β coefficients (that is, they are standardized), and arrow thickness is proportional to effect size. Solid black arrows indicate positive β coefficients. Solid arrows entering or leaving secondary school measures are in grey to indicate that they are not accurate estimates of the true effect in the non-selected population. No paths were found which were significant and had negative β coefficients. When interpreting path models, it should be remembered that any analysis towards the right of the diagram takes account of prior effects occurring to the left of the diagram. For this figure, that means, for instance, that the effect of A-level points on Finals takes into account and is additional to the effect of BMS on Finals mark. Abbreviations: A-level, Advanced level; GCSE, General Certificate of Secondary Education; MRCP(UK), Membership of the Royal College of Physicians of the United Kingdom; PACES, Practical Assessment of Clinical Examination Skills; UCLMS, University College London Medical School.

Figure 3 shows the Academic Backbone path diagram for the 1985 Cohort Study in a similar way to that in Figures 1 and 2. All paths are significant at *P*<0.05. The backbone is particularly clear in this diagram, with only one path that is not directly between successive elements of the diagram.

### Sex and ethnicity effects

Sex and ethnicity effects are described in more detail in the supplementary material (see Additional file 2: Information file). O-level results were complex with a sex × ethnicity interaction, the mean score being similar in white and non-white males, but with white females having a higher score and non-white females having a lower score overall than both male groups. Using simple *t*-tests, we found that females performed better overall than males, and whites slightly better than males. Males had higher A-level grades but in the path analysis, they underperformed at BMS, performed equivalently at Finals, and were more likely to be on the Specialist Register. Non-white participants performed less well at Finals, but were equally likely to be on the Specialist Register.

### The 1980 Cohort Study

The 1980 Cohort Study was a study of selection, with data available for applicants and entrants. Of 1,362 individuals in the study, 517 (38.0%) were accepted by a UK medical school in 1981 [36], and a further 74 in 1982, making 591 overall. Medical schools provided separate information on performance in the first and second BMS years on a four-point scale (1 = failed, withdrew, repeated year, etc.; 2 = Passed after resits; 3 = Satisfactory; 4 = Distinction). For the 565 students in their first year, the percentages in the four categories (1, 2, 3, and 4) were 3.5%, 14.9%, 75.2%, and 6.4%, respectively), and for the 551 second year students, these were 2.5%, 13.2%. 74.8%, and 9.4%, respectively. Performance at Finals was available only for the 330 students who took examinations at the University of London, and was similar in structure to that for the 1985 Cohort Study. At follow-up in 2009, 407 doctors were known to have qualified, having registered with the GMC, and of these, 168 were on the GP Register, 195 were on the Specialist Register, 44 were on neither Register, and none were on both.

Of the 1,362 applicants, 518 (38.0%) were female, with 39.3% of the 591 entrants being female. Ethnicity was not collected directly in the survey of entrants, but was collected for the 335 participants who responded to the final year questionnaire. For other applicants, surnames were coded as European or non-European, a method with good validity [42], and in the present data there was 94.9% agreement with self-classification. Overall, 318 (23.3%) of the 1,362 applicants were from ethnic minorities, as were 16.1% of the 591 entrants.

Three or more A-level results were available for 1,220 applicants and 587 entrants, and six or more O-level results were available for 1,191 applicants and 555 entrants. For the best three A-level grades (excluding General Studies, as usual), the mean ± SD was 19.8 ± 6.74 (Median 20), with 7.5% of the 1,220 candidates scoring the maximum of 30 points, and for 587 entrants the mean score was 24.2 ± 4.51 (median 24), with 15.0% of entrants scoring 30 points.

### Reliabilities

No reliability measure is available for the BMS examinations. The total mark at the Finals examination, which comprised 25 separate standardized individual marks from various papers, gave a Cronbach’s α of 0.913 based on 244 candidates.

### Correlations between academic measures

Table 5 shows the correlations in entrants to medical school between the six measures constituting the Academic Backbone: O-levels, A-levels, performance in medical school BMS and clinical examinations, and being on the Specialist Register. Being on the Specialist Register did not correlate with the other measures, but otherwise the correlations were all positive and statistically significant. Correlations in the lower triangle show values corrected for right-censorship and for categorical variables.

**Table 5:** Correlations in the 1980 Cohort Study

	**Mean GCSE/O-level grade**	**Best three A-levels**	**First year BMS outcome**	**Second year BMS outcome**	**Finals outcome**	**On Specialist Register**
	**Continuous censored**	**Continuous censored**	**Ordinal**	**Ordinal**	**Continuous**	**Binary**
Mean GCSE/O-level grade	1	**0.406**	**0.192**	**0.209**	**0.175**	-0.022
		***P*** **<0.001**	***P*** **<0.001**	***P*** **<0.001**	***P *** **= 0.002**	NS
		**n = 562**	**n = 542**	**n = 528**	**n = 313**	n = 391
Best three A-levels	**0.438 ± 0.035 (0.358 to 0.503)**	1	**0.190**	**0.249**	**0.307**	0.045
			***P*** **<0.001**	***P*** **<0.001**	***P*** **<0.001**	NS
			**n = 562**	**n = 548**	**n = 328**	n = 403
First year BMS outcome	**0.226 ± 0.041 (0.130 to 0.298)**	**0.235 ± 0.042 (0.150 to 0.318)**	1	**0.416**	**0.215**	0.005
				***P*** **<0.001**	***P*** **<0.001**	NS
				**n = 548**	**n = 329**	n = 397
Second year BMS outcome	**0.235 ± 0.047 (0.148 to 0.338)**	**0.293 ± 0.054 (0.174 to 0.379)**	**0.512 ± 0.038 (0.434 to 0.582)**	1	**0.359**	0.080
					***P*** **<0.001**	NS
					**n = 329**	n = 399
Finals outcome (London only)	**0.182 ± 0.051 (0.081 to 0.280)**	**0.323 ± 0.052 (0.229 to 0.426)**	**0.244 ± 0.064 (0.107 to 0.357)**	**0.410 ± 0.052 (0.304 to 0.511)**	1	0.084
						NS
						n = 261
On Specialist Register	-0.031 ± 0.062 (-0.146 to 0.090)	0.054 ± 0.063 (-0.064 to 0.176)	0.007 ± 0.079 (-0.142 to 0.156)	0.119 ± 0.077 (-0.052 to 0.255)	0.104 ± 0.074 (-0.038 to 0.250)	1

*Abbreviations*: A-level, Advanced level; BMS, Basic medical sciences; GCSE, General Certificate of Secondary Education; MCMC, Markov Chain Monte Carlo; NS, not significant; O-level, Ordinary level.

^a^Correlations in entrants to medical school between measures of academic and professional attainment attainment in the 1980 Cohort study.

^b^Correlations above the diagonal are simple Pearson correlations (for example point-biserials, φ correlations. where a measure is binary or ordinal), and have different n values for various reasons.

^c^Correlations shown in bold are significant at *P*<0.05.

^d^Correlations in the lower triangle were calculated taking account of censoring, and for ordinal values are equivalent to tetrachoric/polychoric correlations.

^e^Values are mean ± SD (95% CI) unless otherwise stated, and the 95% confidence intervals are the result of the final 2000 MCMC steps in a chain of 5000.

### The academic backbone

**Figure 4:**
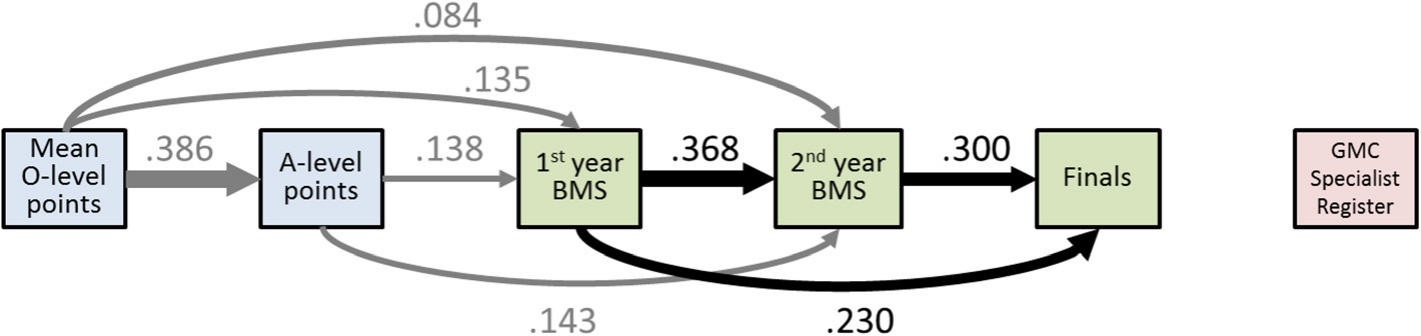
**The Academic Backbone in the 1980 Cohort Study.** This figure, and Figures 1, 2, 3, and 5, which show path analyses of the Academic Backbone in the various cohorts, have the same structures and conventions, and are also very similar to the figures in the additional material (see Additional files). Pale blue boxes indicate measures obtained prior to medical school, usually at secondary school, pale green boxes indicate performance at medical school, and pale purple boxes indicate post-graduate performance. The path model was fitted using multiple regression, each variable being regressed on all variables to its left (that is, causally prior), using backwards regression, variables being eliminated sequentially until all remaining variables were significant with *P*<0.05. Path coefficients are shown as β coefficients (that is, they are standardized), and arrow thickness is proportional to effect size. Solid black arrows indicate positive β coefficients. Solid arrows entering or leaving secondary school measures are in grey to indicate that they are not accurate estimates of the true effect in the non-selected population. No paths were found which were significant and had negative β coefficients. When interpreting path models, it should be remembered that any analysis towards the right of the diagram takes account of prior effects occurring to the left of the diagram. For this figure, that means, for instance, that the effect of 1^st^ year BMS marks on Finals takes into account and is additional to the effect of 2^nd^ year BMS marks on MRCP(UK) Part 1 mark. Abbreviations: A-level, Advanced level; GCSE, General Certificate of Secondary Education; MRCP(UK), Membership of the Royal College of Physicians of the United Kingdom; PACES, Practical Assessment of Clinical Examination Skills; UCLMS, University College London Medical School.

Figure 4 shows the Academic Backbone path diagram for the 1980 Cohort Study in a similar way to that in Figures 1, 2 and 3. All paths are significant at *P*<0.05. The backbone is clearly shown, with direct and indirect paths from O-levels to Finals. Although there are no links to being on the Specialist Register, it is of interest is that once sex differences are taken into account (see Additional file 2: Information file), a link is present between second year BMS results and being on the Specialist Register.

### Sex and ethnicity effects

Sex and ethnicity effects are described in detail (see Additional file 2: Information). Male participants performed less well at O-level, and then less well at BMS and Finals, although only the effect on Finals was significant in the path model. Males were more likely to be on the Specialist Register after taking earlier performance into account in the path model. Non-white participants showed no differences from white participants in the simple analyses or the path model.

### The Westminster Cohort Study

The Westminster Cohort Study is the oldest of the five cohort studies, and it was not a selection study. Although not particularly large, the study had both an aptitude test (the full AH5) and measures of performance in house jobs (PRHO posts). Performance on MRCP(UK) Part 1 was available, but the number of participants taking it was low, giving relatively little power. Performance on the clinical school was available as a four-point rating (4 = Distinction; 3 = Pass on Finals first time; 2 = Finals passed after resit; 1 = Failed Finals), with the numbers in the four groups being 9, 428, 42, and 7, respectively. Consultants rated PRHOs on a four-point scale (4 = Outstanding; 3 = Good; 2 = Satisfactory; 1 = Inadequate), the modal number of consultants being four (two medicine, two surgery). Of 505 participants in the original study, at follow-up in 2009, 458 were on the Medical Register, 230 on the Specialist Register, 197 on the GP Register, 6 on both, and 37 on neither.

Of the 505 original participants, 123 (24.5%) were female. Self-declared ethnicity was not assessed in the study, but only 12 (2.4%) of the participants had non-European surnames, a measure that correlates well with self-described ethnicity [42]. There were 501 participants who had gained three or more A-level results. For the best three A-level grades (excluding General Studies, as usual), the mean ± SD was 24.4 ± 4.30 (median 24), with 14.6% of entrants scoring the maximum 30 points. Only 106 participants had taken MRCP(UK), and because these were at different diets (and hence pass marks were different), results at the first attempt at Part 1 are presented in the standard four categories of 4 = Good Pass (33.0%), 3 = Pass (19.8%), 2 = Fail (42.5%), and 1 = Bad Fail (4.7%).

### Reliabilities

No reliability measure is available for the clinical examinations. However for the house job ratings, considering participants four or more separate ratings, the reliability based on four ratings was 0.609.

### Correlations between academic measures

Table 6 shows the correlations between the AH5 score, A-levels, clinical examination performance, PRHO ratings, MRCP(UK) performance, and being on the Specialist Register. Sample sizes are small in some cases, and binary and ordinal measures also lack power. However, there are clear correlations from AH5 scores to PRHO ratings, and also to being on the Specialist Register. The numbers taking MRCP(UK) are probably too small to come to any reasonable conclusions. Correlations in the lower triangle show values corrected for right-censorship and for categorical variables.

**Table 6:** Correlations in the Westminster Cohort Study

	**AH5 score**	**Best three A-levels**	**Clinical performance**	**PRHO ratings**	**MRCP(UK) Part 1**	**On Specialist Register**
	**Continuous**	**Continuous censored**	**Ordinal**	**Continuous censored**	**Ordinal**	**Binary**
AH5 score	1	**0.281**	**0.155**	0.074	-0.162	**0.185**
		***P*** **<0.001**	***P *** **= 0.001**	NS	NS	***P*** **<0.001**
		**n = 501**	**n = 486**	n = 408	n = 106	**n = 458**
Best three A-levels	**0.299 ± 0.041 (0.223 to 0.377)**	1	**0.146**	0.078	0.098	**0.190**
			***P *** **= 0.001**	NS	NS	***P*** **<0.001**
			**n = 483**	n = 405	n = 105	**n = 454**
Clinical performance	**0.209 ± 0.065 (0.079 to 0.336)**	**0.202 ± 0.057 (0.091 to 0.312)**	1	**0.175**	0.174	**0.126**
				***P*** **<0.001**	NS	***P *** **= 0.008**
				**n = 395**	n = 104	**n = 447**
PRHO ratings	0.069 ± 0.048 (-0.037 to 0.163)	0.086 ± 0.054 (-0.020 to 0.190)	**0.260 ± 0.063 (0.141 to 0.400)**	1	0.093	0.076
					NS	NS
					n = 83	n = 372
MRCP(UK) Part 1	-0.154 ± 0.101 (-0.351 to 0.036)	0.138 ± 0.107 (-0.079 to 0.346)	0.302 ± 0.143 (-0.004 to 0.562)	0.124 ± 0.110 (-0.105 to 0.323)	1	**0.261**
						***P *** **= 0.009**
						**n = 98**
On Specialist Register	**0.229 ± 0.061 (0.109 to 0.348)**	**0.245 ± 0.062 (0.108 to 0.353)**	**0.215 ± 0.077 (0.065 to 0.363)**	0.096 ± 0.069 (-0.042 to 0.231)	**0.325 ± 0.129 (0.053 to 0.572cpa)**	1

*Abbreviations*: A-level, Advanced level; BMS, Basic medical sciences; GCSE, General Certificate of Secondary Education; MCMC, Markov Chain Monte Carlo; NS, not significant; O-level, Ordinary level; PRHO, Pre-Registration House Officer.

^a^Correlations in entrants to medical school between measures of academic and professional attainment in the Westminster Cohort study.

^b^Correlations above the diagonal are simple Pearson correlations (for example, point-biserials, φ correlations, where a measure is binary or ordinal), and have different N values for various reasons.

Correlations shown in bold are significant at *P*<0.05.

Correlations in the lower triangle were calculated taking account of censoring, and for ordinal values are equivalent to tetrachoric/polychoric correlations.

^e^Values are mean ± SD (95% CI) unless otherwise stated, and the 95% confidence intervals are the result of the final 2000 MCMC steps in a chain of 5000.

### The Academic Backbone

**Figure 5:**
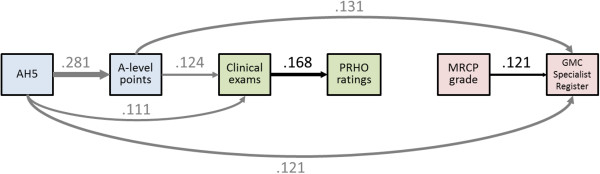
**The Academic Backbone in the Westminster Cohort Study.** This figure, and Figures 1, 2, 3, and 4, which show path analyses of the Academic Backbone in the various cohorts, have the same structures and conventions, and are also very similar to the figures in the additional material (see Additional files). Pale blue boxes indicate measures obtained prior to medical school, usually at secondary school, pale green boxes indicate performance at medical school, and pale purple boxes indicate post-graduate performance. The path model was fitted using multiple regression, each variable being regressed on all variables to its left (that is, causally prior), using backwards regression, variables being eliminated sequentially until all remaining variables were significant with *P*<0.05. Path coefficients are shown as β coefficients (that is, they are standardized), and arrow thickness is proportional to effect size. Solid black arrows indicate positive β coefficients. Solid arrows entering or leaving secondary school measures are in grey to indicate that they are not accurate estimates of the true effect in the non-selected population. No paths were found which were significant and had negative β coefficients. When interpreting path models, it should be remembered that any analysis towards the right of the diagram takes account of prior effects occurring to the left of the diagram. For this figure, that means, for instance, that the effect of AH5 on Clinical exams takes A-level points into account and is additional to the effect of A-level points on Clinical exams. Abbreviations: A-level, Advanced level; GCSE, General Certificate of Secondary Education; MRCP(UK), Membership of the Royal College of Physicians of the United Kingdom; PACES, Practical Assessment of Clinical Examination Skills; UCLMS, University College London Medical School.

Figure 5 shows the Academic Backbone path diagram for the Westminster Cohort Study, in a similar way to that f Figures 1, 2, 3, and 4. All paths were significant at *P*<0.05. The backbone is clearly shown, from AH5 through to being on the Specialist Register. Further information on analyses of sex and ethnicity is available (see Additional file 2: Information), although the number of ethnic minority participants is very low.

### Sex and ethnicity effects

Sex and ethnicity are described in detail (see Additional file 2: Information). Male participants did not show differences in performance on simple tests, but in the path model, after taking earlier performance into account, males had rather lower PRHO ratings than did females, and were more likely to be on the Specialist Register. Non-white participants performed slightly less well than white participants on the AH5 test, but otherwise showed no difference in overall performance.

## Discussion

The five cohort studies analyzed in this study considered students who entered UK medical schools in 1973 to 1980 (the Westminster Study), 1981, 1986, and 1991 (the 1980, 1985, and 1990 Cohort Studies), and 2003 to 2005 (the UCLMS Cohort Study). Many things have changed during that time, with the proportion of women entrants rising (24.5%, 39.3%, 45.2%, 50.5%, and 60.5%, respectively) and the proportion of ethnic minority entrants also rising (2.4%, 16.1%, 17.1% , 25.3%, and 52.9%, respectively), although some of these differences may in part reflect different sampling frames. The proportion of entrants with maximum AAA grades has also risen (14.6%, 15.0%, 18.7%, 21.3%, and 65.2%, respectively), gently at first and then sharply with the new millennium (and current figures suggest the figure is between 80% and 90%, although the problem has been ameliorated somewhat by the introduction of A* grades at A-level). With so much change, it is interesting to assess to what extent commonalities exist across cohorts.

Underlying the various analyses is the theoretical concept of the Academic Backbone, the idea that in medical education, current learning and achievement is critically dependent upon achievement at earlier stages. Visually, the backbone can be seen in all of Figures 1, 2, 3, 4, and 5 although the five studies do differ in their sample sizes, making the studies differ in their power for detecting particular effects, with the 1990 sample being the largest (and as Figure 2 shows, the one in which most effects are identified). The Academic Backbone is particularly visible in the most detailed study, the UCLMS Cohort, which has simple correlations of 0.75, 0.52, 0.73, and 0.83 between the marks in years 1 and 2, years 2 and 3, years 3 and 4, and years 4 and 5, respectively, suggesting a robust prediction of any one year’s performance by the previous year’s performance. Correlations of BMS with clinical years are also solid in the other studies (0.39, 0.41, and 0.41 for the 1990, 1985, and 1980 Cohort studies). MRCP(UK) performance and being on the Specialist Register also show consistent correlations with undergraduate performance, most obviously in the UCLMS cohorts. In all the cohorts, it is clear that at almost every stage of attainment, there are causal links back to attainment at earlier stages. A similar result to this was found in a recent meta-analysis of US medical school attainment as a predictor of residency outcome. Averaging across studies, performance at residency (National Board of Medical Examiners (NBME) 3, in-training examinations and Licensing examinations) correlated at 0.596 (weighted mean) with measures of clinical performance in medical school (NBME 2 and United States Medical Licensing Examination 2 (USMLE2)), and 0.515 (weighted correlation) with measures of BMS performance (NBME 1 and USMLE 1) [43], confirming the nature of the Academic Backbone from medical school through to residency.

The correlation of performance at later stages in medical training with earlier stages does not just take place in medical school itself, but begins earlier than that, with evidence of later achievement in medical school (and beyond) correlating not only with A-level results, but also with GCSE/O-levels. Equivalent results have been found in the USA, where USMLE 1, 2, and 3 examinations are predicted by performance on the Medical College Admission Test (MCAT), which is taken prior to entry into medical school (meta-analytic correlations: 0.60, 0.38, and 0.43, respectively) [44].

Of particular interest in the present study are correlations with A-levels. Remembering that correlations, even those corrected for right-censoring, are within entrants and not within applicants in general, it is clear that A-levels predict medical school performance with an average correlation of 0.24, predict MRCP(UK) results with a slightly lower average correlation of 0.21, and predict being on the Specialist Register with an average correlation of 0.15. Elsewhere, we estimated construct validities for the measures of educational attainment in these five studies and the much larger UKCAT-12 study of performance in the first BMS year [8], and found that construct validities were substantially higher than simple correlations, explaining up to 65% of the variance in BMS first year performance [8].

Although we have identified evidence for the Academic Backbone in terms of correlations between attainment at different educational stages, that is not to imply merely that it is examination performance at one stage that predicts examination performance at a later stage. A better interpretation is that it is the structure of knowledge that enables people to perform at a particular level in assessment 1, and that earlier structure of knowledge is the platform on which a more sophisticated structure of knowledge is built at stage 2, and itself provides a platform for achieving in assessment 2. Neither should it be construed that the backbone is about isolated nuggets of information ('factoids’ as they have been called) that are merely learned, but instead it is systems of information, categories of knowledge, metaphors, patterns and relationships, interconnected images and ideas that are acquired, all of which make it possible to understand new information, integrate it with existing ideas, and position it in memory in a way that it can then be accessed easily and triggered in the right circumstances. The end result is the accumulation of what in general can be referred to as 'cognitive capital’, or more specifically in the present context can be described as 'medical capital’; that set of knowledge, theories, experience, understanding and skills that comprise successful medical practice.

**Figure 6:**
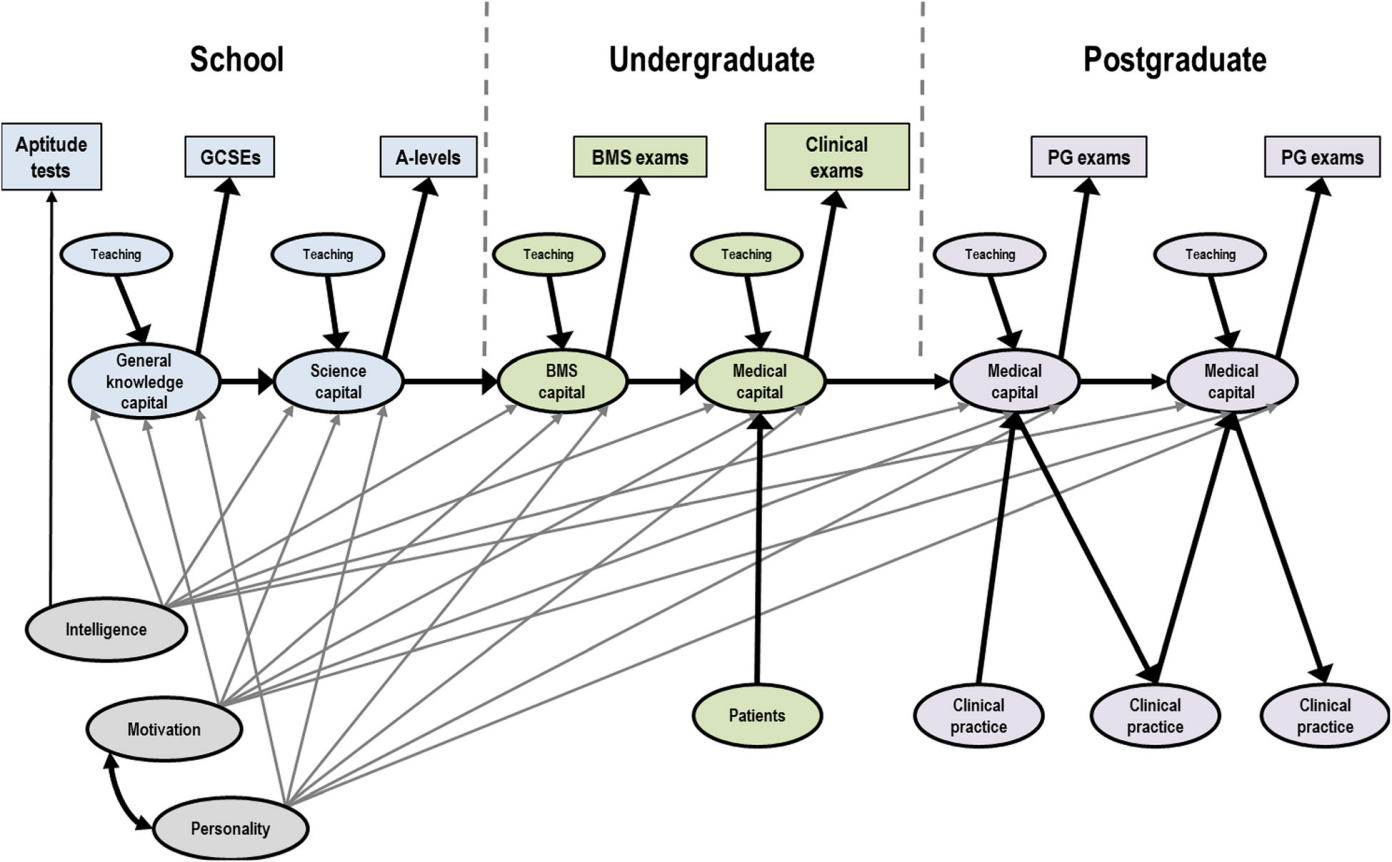
A more detailed model of the Academic Backbone, showing the relation of the various measures of attainment (top row) with the acquisition of medical capital, aided by teaching, patients, and clinical practice, with influences from intelligence, motivation, and personality.

Figures 1, 2, 3, 4, and 5 show the causal relations between the various measured variables. In reality, many of those measured variables are indicators of other, underlying, latent variables, and Figure 6 shows a more comprehensive model of the probable inter-relations. The rectangular boxes show the same measured variables as in Figures 1, 2, 3, 4, and 5, while the ellipses show variables that have not been measured here, but in principle could be measured. The main backbone is now of educational capital, starting at primary school, where generic cognitive capital (not shown) is acquired during the learning of reading, writing, arithmetic, and other basic skills. By secondary education, a wide range of 'general knowledge capital’ is acquired, helped particularly by specific teaching, in subjects such as history, geography, languages, sciences, and art, all of which are then assessed by GCSEs. The general knowledge capital is then the underpinning for more specific 'science capital’, through subjects such as chemistry and biology, which are assessed by A-levels. At medical school, 'BMS capital’ is acquired, again with the help of teaching, and built on the foundations of the science capital studied at A-level. In the clinical years of medical school, 'medical capital’ proper begins to be acquired, aided once again by teaching, but also assisted by experience of patients and patient care, with assessment in the form of clinical examinations. Medical capital continues to be acquired during post-graduate training, but now, although there is still teaching, there is also extensive clinical practice, which aids the acquisition of medical capital while the medical capital improves the quality of subsequent clinical practice. Medical capital not only grows during medical training but can also diminish, through spontaneous decay and forgetting, or through earlier knowledge becoming out of date, so that the trajectory of capital growth is not always smooth [45]. Medical capital during the post-graduate years is assessed by various post-graduate examinations at different stages. Throughout training, from primary and secondary school to post-graduate studies, the acquisition of medical and other forms of capital is enhanced by intelligence, appropriate motivation and appropriate personality. Pure intellectual aptitude tests (shown at the top left) are indicators primarily of intelligence, as shown at the bottom of Figure 6, with intelligence as such being assessed by aptitude tests (and perhaps also general knowledge capital). Aptitude tests can also assess science knowledge, in which case they are also influenced by science capital (link not shown in Figure 6). The path model of Figure 6 predicts that there will be positive correlations between all of the measures in the top row (in rectangular boxes).

The concept of the Academic Backbone is supported empirically by data at all stages of medical training, from secondary school-level achievement through to post-graduate examinations. Of importance is that it is not merely achievement at an immediately previous stage that matters, but, as the path diagrams show, achievement also at many earlier stages. Once an examination is passed, then the learning that preceded that examination is not erased and the next stage is not coped with in isolation. An imprint remains of the learning that contributed to the performance in that earlier examination, and this continues to provide building blocks and scaffolding for what is to follow, in the form of medical capital, which often may be of utility many years later. That is presumably the reason why it is not merely passing an examination which matters, but how well it is passed, with those on the borderline subsequently having greater difficulties than those who passed an examination at a higher level [26].

The role of A-levels in UK medical student selection has long been debated, with there being many claims that high achievement at A-level has little or no predictive value for subsequent medical attainment. The backbone studies clearly show that is not the case. A-levels, despite an ever-narrowing range of marks due to grade inflation and ceiling effects, continue to predict performance significantly throughout medical school, and often onto post-graduate examinations. There is therefore no simple 'threshold’ above which higher attainment at A-level makes little difference to outcome. Rather, higher is always better, a conclusion reached in another educational context by other researchers, with much larger sample sizes [46].

Four of the cohort studies (UCLMS, 1990, 1985, and 1980) included GCSE and/or O-level grades, and in each case it is apparent that these examinations, typically taken at the age of 15 to 16 years, may be useful in selection not only because they anticipate and predict A-level attainment, but because in all the four studies they are predictive of medical school performance, both in BMS (but particularly at the clinical stages), and even, in the 1990 Cohort Study, performance in both the written and clinical examinations in MRCP(UK). That may seem surprising as the science content of A-levels is usually deeper and more extensive than that of GCSEs/O-levels. A possibility is that the predictive value of GCSEs/O-levels comes from the breadth of material that is learned, including in the arts and humanities, all of which can help to inform an understanding of clinical medicine at a broader level than the merely technical.

A controversial topic in student selection is the role of aptitude tests such as UKCAT, BMAT, and Graduate Medical School Admissions Test (GAMSAT) [3]. Most pure aptitude tests are, in all but name, intelligence tests, and two of the cohort studies included the AH5 test, one as a full test and the other as an abbreviated version. In the 1990 Cohort Study, the aAH5 showed no predictive effect of medical school performance (r = 0.05 and 0.06), but it did show correlations with MRCP(UK) (0.13, 0.27 and 0.19). By contrast, in the Westminster Cohort Study, the AH5 seemed to predict undergraduate clinical performance (r = 0.21), but had a negative correlation with MRCP(UK) Part 1 (r = -0.15). Taken overall, it seems doubtful that this test of aptitude is predicting consistently, and certainly the AH5 does not predict as strongly as A-levels or even GCSEs.

Most of the measures in the Academic Backbone are measures of educational and professional attainment, and thus are examinations. A common criticism of the use of examinations in selection and for professional progression is that being good at examinations only predicts the ability to be good at further examinations. However, that argument ignores the content of medical school and professional examinations. It might be argued that examinations such as clinical Finals or MRCP(UK) are nothing but arbitrary tests of irrelevant knowledge, but a content appraisal of the questions makes that unlikely in the extreme. It may be that being a good doctor does not require one to know all of the material included in Finals and post-graduate examinations, but it would seem to be a difficult and perverse argument that clinical knowledge is not only irrelevant to medical practice, but also that a lack of such knowledge makes for better clinical practice. Given the careful blueprinting of examinations and their obvious face validity (and for examples of MRCP(UK) questions see a previous study [47]), we suggest that passing examinations such as those discussed here is important for being a good doctor, and those who have difficulty in attaining such clinical knowledge will probably be less good doctors. Knowledge is generally preferable to ignorance, and clinical knowledge underpins clinical practice.

A measure we have included throughout, mainly because of its ready availability via the GMC LRMP, is being on the Specialist Register. It is not strictly part of the Academic Backbone, but in the four studies where data for it were available, it was predicted by earlier academic measures. However, it was also clearly behaving differently to the other measures, as is well shown in the analyses of sex and ethnicity described below. Because being on the Specialist Register is a key professional outcome for those wishing to work in hospitals, understanding its correlates is important. Overall, there was, inevitably, a very strong negative correlation between being on the Specialist Register and being on the GP Register, with presence on the Specialist Register also being a marker for being in hospital practice rather than general practice. As a result, an important issue concerns the extent to which the measures of the Academic Backbone would predict performance on the Member of the Royal College of General Practitioners (MRCGP) examination. The UCLMS cohort is currently taking MRCGP, and we hope to report formal analyses at a later date, but a preliminary look suggests that GCSEs, A-levels, and medical school assessments do predict performance at MRCGP in a similar fashion to the way they predict MRCP(UK).

The only formal post-graduate assessment we have looked at to date is the MRCP(UK) examinations, and that reflects the fact that we had access to results of MRCP(UK). Clearly, it is necessary to extend our results to other specialties, and it is possible that performing well in post-graduate examinations in, say, surgery, psychiatry, or public health, requires fundamentally different skills, and that correlations with the backbone will be much lower than for post-graduate examinations in internal medicine. Having said that, we find it unlikely on theoretical grounds, but we hope that data will become available for testing this.

Sex differences in performance were found in all of the studies, with males tending to underperform at GCSEs/O-levels, to overperform somewhat at A-levels, and then to underperform once more in medical school examinations. Although the results are not entirely consistent across the cohorts, the male underperformance was also shown in the very large UKCAT-12 study [48].

Perhaps most striking is that in almost all of the studies, men were more likely to be on the Specialist Register, irrespective of earlier undergraduate and post-graduate performance. Although specialist practice does tend to be more academic, this is unlikely to explain the male predominance, and instead it is probably necessary to consider either factors differentially related to motivation or a drive for career success, or a host of sociological, familial, and personal reasons that mean female doctors may not enter the stricter, less flexible confines of hospital practice, and instead would enter general practice [49].

Ethnic differences in performance show some similarities to sex differences. Like men, those from ethnic minorities, underperformed at GCSE/O-level relative to A-levels, and particularly underperformed at clinical rather than BMS assessments. This in part is mediated via the link between GCSEs/O-levels and clinical performance, but seems to show an effect in addition to that. Although ethnic minority underperformance is widespread in medicine [15], of particular interest is that the two earliest studies show little effect. In part, the lower proportion of ethnic minority participants may mean there is less statistical power to detect ethnicity effects, with the Westminster Study in particular having a very low proportion of ethnic minority participants, and such low proportions reflecting the secular changes that have occurred in medical school entry. Lack of power probably cannot explain the absence of ethnic differences in the 1980 Cohort, as with an estimated effect size of 0.42 (based on the meta-analysis of Woolf *et al*. [15]), an α of 0.05 and sample sizes of 95 and 495 in the two groups, there was a power of 98% for detecting an effect. One possibility, as we have suggested elsewhere, is that the social networks of minority and non-minority students are clustered ethnically, and that contributes to poorer performance [50]. It may be that in the past, when there were relatively fewer ethnic minority medical students in medical schools, minority students were more integrated into the social network, which helped to minimize ethnic differences in performance.

## Conclusions

The correlations shown here, between attainment in secondary schooling, undergraduate medical education, and post-graduate medical education, strongly support the existence of the Academic Backbone, with effects spanning many years. The Academic Backbone can be conceptualized in terms of the development of the development of ever more sophisticated underlying structures of knowledge, 'cognitive capital,’ and 'medical capital’, with the latter being acquired during education, and through clinical experience with patients. The Academic Backbone provides strong support for using measures of educational attainment, such as A-levels, in student selection.

## Abbreviations

aAH5: Abbreviated AH5; AH5: Group test of General Intelligence; A-level: Advanced level; BMAT: BioMedical Admissions Test; BMS: Basic medical sciences; BOF: Best-of-five; GCSE: General Certificate of Secondary Education; GMC: General Medical Council; GP: General practitioner; IRT: Item-response theory; MCAT: Medical College Admissions Test; MCMC: Markov Chain Monte Carlo; MRCP(UK): Membership of the Royal College of Physicians of the United Kingdom; MTF: Multiple true-false; NHS: National Health Service; nPACES: New Practical Assessment of Clinical Examination Skills; O-level: Ordinary Level examinations; OSCE: Objective structured clinical examination; PACES: Practical Assessment of Clinical Examination Skills; PRHO: Pre-Registration House Officer; RFUCMS: Royal Free and University College Medical School; SD: Standard deviation; UCAS: Universities and Colleges Admissions Service; UCCA: Universities Central Council on Admissions; UCL: University College London; UCLMS: University College London Medical School; UCMSM: University College and Middlesex School of Medicine; UKCAT: United Kingdom Clinical Aptitude Test; UMDS: United Medical and Dental Schools (Guy’s and St. Thomas’s).

## Competing interests

The author(s) declare that they have no competing interests.

## Authors’ contributions

The idea for the study came from ICM, after discussions with KW and CD. ICM and EP had collaborated on several follow-ups of the 1980, 1985, and 1990 cohort studies. KW’s research on the UCLMS cohort was supervised by JD and ICM. JD and ICM have collaborated on linking data from MRCP(UK), of which JD is Medical Director, with other databases. Statistical analyses were carried out by ICM in collaboration with CD. The first draft was written by ICM, and KW, CD, EP, and JD all contributed to revisions and to the final draft. All authors read and approved the final manuscript.

## Supplementary Material

Additional file 1 Sex and ethnicity effects in the five longitudinal cohort studies. Click here for file

Additional file 2 The MCMC method for correcting correlations for right- and left-censoring. Click here for file
