# Supplementary material for: The Academic Backbone: longitudinal continuities in educational achievement from secondary school and medical school to MRCP(UK) and the specialist register in UK medical students and doctors
Source: BMC Med. 2013 Nov 14;11:242. doi: 10.1186/1741-7015-11-242 (PMC3827330; doi:10.1186/1741-7015-11-242)

Additional Information File

Sex and ethnicity effects in the five longitudinal cohort studies

The UCLMS Cohort Study.

60.6% of 715 UCLMS medical students were female, a proportion that was not significantly different in White (209/337; 62.0%) and non-White students (224/378; 59.3%; chi-square = .568, 1df, p=.451). Table 3 summarises the performance of male and female students from White and non-White groups on the measures in the study, and also shows the results of an analysis of variance. For the GCSE and A-level qualifications, female students had higher grades at GCSE (but not A-level). At A-level, non-White students had *higher* A-level grades, but despite that, performed significantly *less* well on all five measures of medical school performance. Repeated measures ANOVA found no difference in the relative performance of males and females or white and non-white students on written versus OSCE/performance tests. Males performed better on BMS exams, and females on clinical exams, and repeated measures ANOVA found a highly significant exam type x sex interaction (p<.001), a significant exam type x ethnicity interaction (p<.001), and a just significant exam type x sex x ethnicity interaction (p=.042). In the MRCP(UK) exams, women performed better at Part 1, but otherwise there were no significant differences, although Ns were small for Part 2 and PACES.

The path model for the academic backbone, shown in figure 1, is readily extended to include sex and ethnicity effects (see figure 1b). Males and females perform differently at different stages, with males doing less well at GCSE, but relatively better at A-level, taking GCSE performance into account. Males then perform better in BMS exams, but less well in clinical exams, and then better once more in MRCP(UK) Part 1. Non-white students perform less well at BMS after having taken A-levels into account, and then perform even less well at clinical exams, after taking BMS performance into account.

The 1990 Cohort Study.

Analyses in table 2 are similar to those for the UCLMS Cohort Study (table 1). Males and females and White and Non-White participants showed significant differences on a number of measures, with evidence of interaction in only two of the twelve analyses (and those would not reach significance with a Bonferroni correction). Male participants had higher scores on the best three A-level grades, but had lower O-level/GCSE results, and performed less well at Basic Medical Sciences, Finals, were less likely to take MRCP(UK), and performed less well at Part 2 Clinical. However they were more likely to be on the GMC Specialist Register. In many ways non-White participants showed a similar pattern to that of males, having higher scores at A-level (and had taken more A-levels), but had lower scores at O-level/GCSE, performed less well at Basic Medical Sciences and Finals, and performed less well at all three parts of MRCP(UK). They were however equally likely as White participants to be on the Specialist Register.

Not all medical graduates choose to take MRCP(UK), and the simple analyses of table 2 suggest that while females are more likely to take the exam, there are no differences in the rate of taking amongst White and non-white participants. However taking MRCP(UK) is significantly correlated with performance at BMS and particularly with performance at Finals. Once BMS and Finals are taken into account, males are still significantly less likely to take MRCP(UK) (logistic

regression, odds ratio -.751, p=.001), but non-White participants are more likely to take MRCP(UK) (odds ratio = 1.358, p=.003).

Figure 2 shows the model of the Academic Backbone from the main paper, with sex and ethnicity effects being added. It can be seen that both male and non-white participants score higher at A-level, but then underperform during medical school. However males then perform relatively better at MRCP(UK) Part 1 (after taking earlier differences into account), whereas non-White participants underperform at MRCP(UK). In part those differences probably reflect differences in those choosing to take MRCP(UK).

The 1985 Cohort Study.

Analyses in table 3 are similar to those for the UCLMS Cohort Study and 1990 Cohort Study (tables 1 and 2). Males and females and White and Non-White participants showed various significant differences, with only one variable suggesting the presence of an interaction. Male participants had higher scores on the best three A-level grades, and non-white participants had lower O-level/GCSE results, and performed less well at Finals. Males were more likely to be on the GMC Specialist Register.

Figure 3 shows the model of the Academic Backbone from the main paper, with sex and ethnicity effects being added. Males underperform at O-level but then score relatively higher at A-level, and underperform during medical school. However males are still more likely to be on the Specialist Register. Ethnicity seems mainly to have an effect at Finals. The apparent discrepancies between table 3 and figure 3, particularly at O-level, reflect the interaction term and how it is treated in the ANOVA, using method 3 for sums of squares, compared with path analysis of figure 3 which only uses main effects.

The 1980 Cohort Study.

Analyses in table 4 are similar to those for the UCLMS Cohort Study and 1990 and 1985 Cohort Studies (tables 1, 2 and 3). There are clear sex differences, with female having higher O-level grades, as well as performing better in first and second year Basic Medical Science examinations..

Figure 4 shows the path model of the Academic Backbone including sex and ethnicity effects. As expected there are clear sex differences, with males underperforming at O-level and again at second year BMS examinations, but despite that they are more likely to be on the Specialist Register. As mentioned in the main text, the inclusion of sex also means that there is a significant link from second year BMS examinatons to being on the Specialist Register, the effect presumably being masked in the simpler analysis of the main text by confounding with sex. Also striking about figure 4 is that there are no effects of ethnicity at all, as was also found in table 4, white and non-white participants performing equivalently at all stages.

The Westminster Cohort Study.

Table 5 summarises the analyses of the various measures in the Westminster Cohort Study by sex and ethnicity. Power is however low, as the proportion of females is much lower than in the other studies, and the proportion of ethnic minority participants is extremely low. However, as figure 5 shows, the path model finds that males underperform on PRHO ratings, and yet, as in the other

studies, are more likely to be on the Specialist Register. Ethnicity has only one effect, minority participants performing less well on the AH5, although given the small sample size the result should be treated with great care.

Additional Table 1. UCLMS Cohort study . Comparison of Male and Female, and White and non-White participants on the various measures. The last three columns show the significance of differences using 2-way analysis of variance. F: Female, M: Male, W: White; NW: Non-White.

Mean or Pct (SD) n n	Male participants		Female participants		ANOVA		
	White	Non-White	White	Non-White	Sex	Ethnicity	Inter-action
Number of GCSEs	10.2 (.98) 108	10.0 (1.2) 134	9.92 (.94) 163	10.1 (1.35) 190	NS	NS	NS
Mean points per GCSE	5.15 (.55) 109	5.25 (.54) 133	5.38 (.44) 160	5.41 (.42) 188	**P<.001 F>M**	NS	NS
Total GCSE points	52.5 (8.4) 108	52.7 (8.1) 133	53.6 (7.1) 159	55.0 (7.8) 188	**P=.011 F>M**	NS	NS
Number of A-levels	3.44 (.66) 120	3.47 (.57) 143	3.49 (.60) 178	3.45 (.69) 216	NS	NS	NS
Mean A-level grade	9.29 (1.09) 120	9.52 (.78) 143	9.27 (1.01) 178	9.49 (.84) 216	NS	**P=.002 NW>W**	NS
Total points for three best A-levels	28.1 (3.4) 120	28.7 (2.2) 143	28.1 (3.0) 179	28.7 (2.4) 216	NS	**P=.005 NW>W**	NS
Overall performance at Medical School	.216 (.915) 127	-.194 (.940) 153	.216 (.798) 208	-.104 (.800) 224	NS	**P<.001 W>NW**	NS
Basic Medical Science exam performance	.242 (1.04) 104	.020 (.946) 134	.034 (.883) 170	-.178 (.896) 202	**P=.009 M>F**	**P=.006 W>NW**	NS
Clinical exam performance	.172 (.950) 126	-.377 (1.09) 153	.245 (.827) 206	-1.05 (.825) 224	**P=.015 F>M**	**P<.001 W>NW**	NS
Written exam performance	.196 (.942) 127	-.136 (.931) 153	1.73 (.808) 208	-.096 (.824) 224	NS	**P<.001 W>NW**	NS
OSCE/practical exam performance	.202 (.816) 127	-.182 (.826) 153	.191 (.726) 208	-.093 (.688) 224	NS	**P<.001 W>NW**	NS
MRCP(UK) attempted	33.6% N=128	34.4% N=154	33.5% N=209	37.5% N=224	NS	NS	NS
MRCP(UK) Part 1 mark	9.58 (8.54) 43	6.31 (10.1) 53	4.66 (10.1) 70	3.53 (10.2) 84	**P=.003 M>F**	NS	NS
MRCP(UK) Part 2 mark	10.74 (10.4) 26	10.4 (9.49) 24	8.87 (9.96) 31	6.47 (7.84) 39	NS	NS	NS
MRCP(UK) PACES mark	4.51 (4.76) 10	5.35 (5.30) 15	6.21 (3.66) 18	3.41 (4.90) 15	NS	NS	NS

Additional Table 2. 1990 Cohort study . Comparison of Male and Female, and White and non-White participants on the various measures in medical school entrants. The last three columns show the significance of differences using 2-way analysis of variance. F: Female, M: Male, W: White; NW: Non-White.

Mean (SD) n or *Pct n*	Male participants		Female participants		ANOVA		
	White	Non-White	White	Non-White	Sex	Ethnicity	Inter-action
aAH5 total score	21.0. (4.32) 257	18.8 (4.25) 123	20.4 (4.31) 268	19.7 (3.77) 97	NS	**P<.001 W>NW**	P=.037
O-level/GCSE results expressed as z-score	.339 (.689) 912	.225 (.884) 343	.625 (.544) 1056	.416 (.757) 285	**P<.001 F>M**	**P<.001 W>NW**	NS
Number of A-levels	3.36 (1.06) 1044	3.49 (1.14) 421	3.30 (1.01) 1187	3.47 (1.02) 333	NS	**P<.001 NW > W**	NS
Mean A-level grade (excluding General Studies)	8.25 (1.46) 1011	8.35 (1.46) 412	8.30 (1.36) 1152	8.11 (1.44) 328	NS	NS	P=.014
Total points for three best A-levels	24.9 (4.86) 1007	25.8 (4.19) 412	24.5 (5.04) 1142	24.8 (4.51) 328	**P=.001 M>F**	**P=.009 NW>W**	NS
Basic Medical Science exam performance	3.51 (.836) 1019	3.43 (.842) 399	3.63 (.748) 1162	3.48 (.839) 326	**P=.049 F>M**	**P<.001 W>NW**	NS
Finals Performance	2.05 (.414) 780	1.96 (.448) 307	2.15 (.392) 941	2.02 (.400) 257	**P<.001 F>M**	**P<.001 W>NW**	NS
MRCP(UK) attempted	29.1% 1044	27.6% 421	35.9% 1187	42.0% 333	**P<.001 F>M**	NS	NS
MRCP(UK) Part 1 mark at first attempt	-.71 (11.1) 280	-2.86 (11.8) 112	-1.75 (10.5) 394	-4.77 (12.4) 130	NS	**P=.004 W>NW**	NS
MRCP(UK) Part 2 Written mark at first attempt	104.8 (18.1) 227	99.6 (15.7) 84	106.5 (18.2) 312	101.0 (16.3)	NS	**P<.001 W>NW**	NS
MRCP(UK) Part 2 Clinical mark at first attempt	17.8 (2.92) 168	16.2 (3.34) 72	18.2 (3.13) 254	17.1 (2.52) 76	**P=.024 F>M**	**P<.001 W>NW**	NS
On GMC Specialist Register (percentage)	58.4% 900	53.0% 336	49.0% 1053	49.2% 275	**P<.001 M>F**	NS	NS

Additional Table 3. 1985 Cohort study . Comparison of Male and Female, and White and non-White participants on the various measures in medical school entrants. The last three columns show the significance of differences using 2-way analysis of variance. F: Female, M: Male, W: White; NW: Non-White.

Mean or *Pct* *(SD) n* *n*	Male participants		Female participants		ANOVA		
	White	Non-White	White	Non-White	Sex	Ethnicity	Inter-action
O-level mean grade	4.24 (.499) 371	4.27 (.623) 90	4.44 (.468) 335	4.16 (.686) 49	NS	**P=.014 W>NW**	P=.002
Total points for three best A-levels	25.3 (3.70) 353	26.3 (3.42) 89	25.0 (4.06) 328	24.7 (4.33) 50	**P=.011 M>F**	NS	NS
Basic Medical Science exam performance	2.65 (.743) 353	2.69 (.819) 94	2.82 (.721) 321	2.64 (.764) 47	NS	NS	NS
Finals Performance	-.093 (.994) 165	-.173 (1.28) 29	.246 (.909) 127	-.312 (.995) 20	NS	**P=.041 W>NW**	NS
On GMC Specialist Register (percentage)	64.2% 310	59.2% 76	47.8% 289	42.1% ()	**P=.002 M>F**	NS	NS

Additional Table 4. 1980 Cohort study . Comparison of Male and Female, and White and non-White participants on the various measures in medical school entrants. The last three columns show the significance of differences using 2-way analysis of variance. F: Female, M: Male, W: White; NW: Non-White.

Mean or *Pct* *(SD) n* *n*	Male participants		Female participants		ANOVA		
	White	Non-White	White	Non-White	Sex	Ethnicity	Inter-action
O-level mean grade	4.19 (.449) 273	4.21 (.449) 66	4.42 (.421)	4.48 (.387)	**P<.001 F>M**	NS	NS
Total points for three best A-levels	24.2 (4.35) 285	23.55 (5.21) 71	24.56 (4.40) 208	24.17 (5.11) 23	NS	NS	NS
Basic Medical Science first year exam performance	2.78 (.583) 272	2.81 (.580) 68	2.92 (.559) 202	3.00 (.522) 23	**P=.026 F>M**	NS	NS
Basic Medical Science first year exam performance	2.85 (.577) 263	2.76 (.609) 66	3.03 (.491) 199	3.00 (.739) 23	**P=.004 F>M**	NS	NS
Finals Performance	-.077 (1.08) 153	-.193 (.980) 129	.124 (.922) 129	.204 (.693) 13	NS	NS	NS
On GMC Specialist Register (percentage)	54.6% 198	55.1% 49	48.6% 146	41.7% 12	NS	NS	NS

Additional Table 5. Westminster Cohort study . Comparison of Male and Female, and White and non-White participants on the various measures in medical school entrants. The last three columns show the significance of differences using 2-way analysis of variance. F: Female, M: Male, W: White; NW: Non-White.

Mean or *Pct* *(SD) n* *n*	Male participants		Female participants		ANOVA		
	White	Non-White	White	Non-White	Sex	Ethnicity	Inter-action
AH5 score	40.8 (7.88) 371	32.9 (6.53) 9	40.1 (7.04) 121	33.0 (5.66) 2	NS	**P=.013 W>NW**	NS
Total points for three best A-levels	24.41 (4.49) 368	21.78 (3.80) 9	24.4 (3.72) 120	25.0 (1.41) 2	NS	NS	NS
Clinical examination performance	2.91 (.396) 366	2.67 (.500) 9	2.91 (.351) 107	3.00 (0) 2	NS	NS	NS
Mean ratings on PRHO jobs	2.93 (.476) 300	2.64 (.408) 7	3.04 (.441) 97	2.75 (.118) 2	NS	NS	NS
MRCP grade	2.90 (.922) 80	2.00 (0) 2	2.58 (1.06) 24	- (-) 0	NS	NS	-
On GMC Specialist Register (percentage)	55.6% 342	71.4% 7	31.1% 106	50.0% 2	NS	NS	NS

Figure captions.

***Additional Figure 1*: UCLMS Cohort Study**. Path analysis of sex and ethnicity effects in addition to the Academic Backbone for the UCLMS cohorts. This figure and Additional Figures 2 to 5 have the same structure and conventions, and are broadly similar to those of figures 1 to 5 in the main text, with some additions. Blue boxes indicate measures obtained prior to medical school, at secondary school, green boxes indicate performance at medical school, and purple boxes indicate postgraduate performance. In addition the red box indicates sex (coded as Male = 1 and Female = 0) and the yellow box indicates ethnicity (codes as Non-White = 1 and White = 0). The path model is fitted using multiple regression, each variable being regressed on all variables to its left (i.e. causally prior), using backwards regression, variables being eliminated sequentially until all remaining variables are significant with $p<.05$. Path coefficients are shown as beta coefficients (i.e. they are standardised), and arrow thickness is proportional to effect size. Solid black arrows indicate positive beta coefficients, and red dashed arrows indicate negative beta coefficients. Arrows between medical school measures and postgraduate measures are in black or dark red. Arrows entering or leaving secondary school measures are in grey or pink to indicate that they are not accurate estimates of the true effect in the non-selected population due to restriction of range (see text). When interpreting path models it should be remembered that any analysis towards the right of the diagram takes account of prior effects occurring to the left of the diagram. For figure 1 that means, for instance, that the effect of BMS marks on MRCP(UK) Part 1 mark takes into account and is additional to the effect of Clinical Marks on MRCP(UK) Part 1 mark. Path coefficients in additional figures 1 to 5 are broadly similar but not identical to path coefficients in main figures 1 to 5, as the addition of extra variables alters the size of the coefficients. Occasionally additional paths are present in the additional figures and these are indicated by blue lines. In the single case where a line is no longer present here but was present in the main figure it is indicated by a pale grey, double line.

***Additional Figure 2*: 1990 Cohort Study.** See caption of Additional Figure 1 for details.

***Additional Figure 3*: 1985 Cohort Study.** See caption of Additional Figure 1 for details.

***Additional Figure 4*: 1980 Cohort Study.** See caption of Additional Figure 1 for details.

***Additional Figure 5*: Westminster Cohort Study**. See caption of Additional Figure 1 for details.

Additional figure 1 (UCLMS).

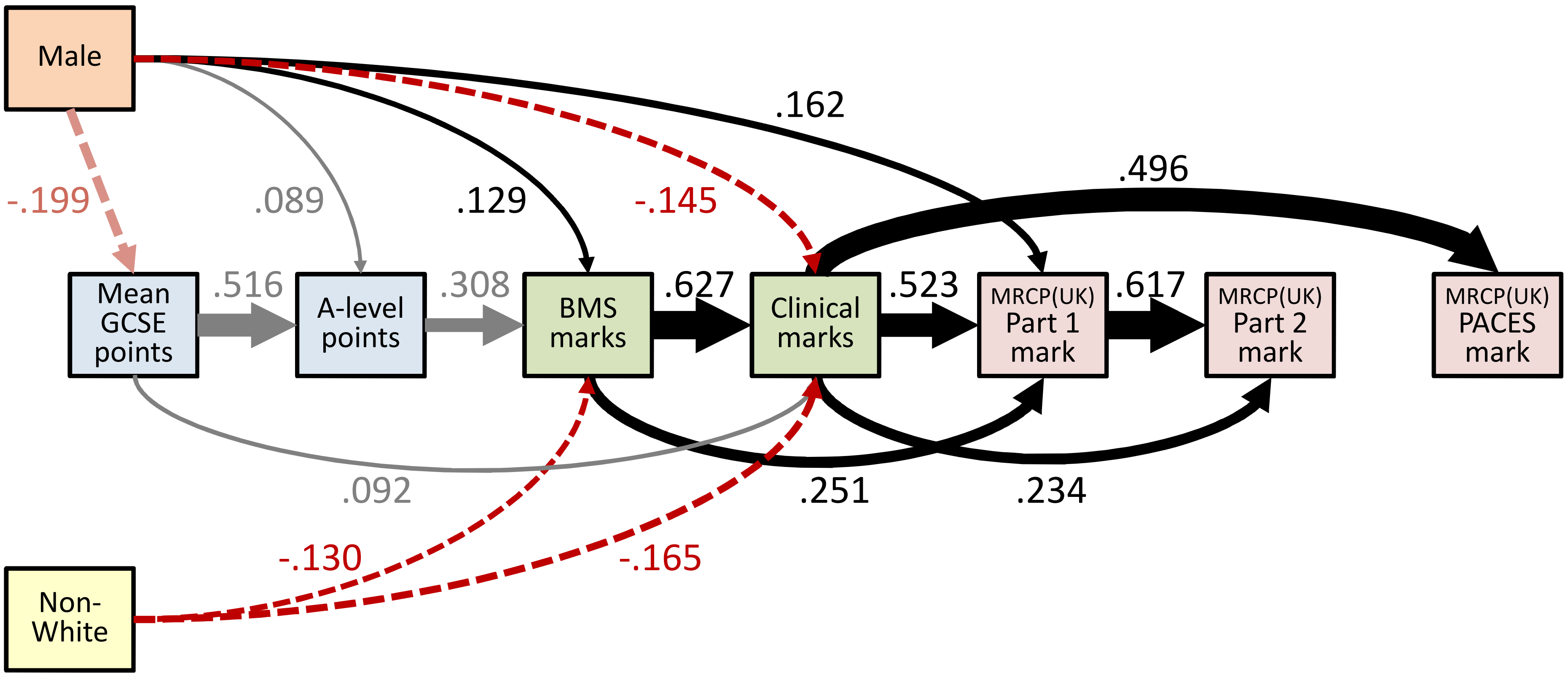

Additional figure 2 (1990 cohort).

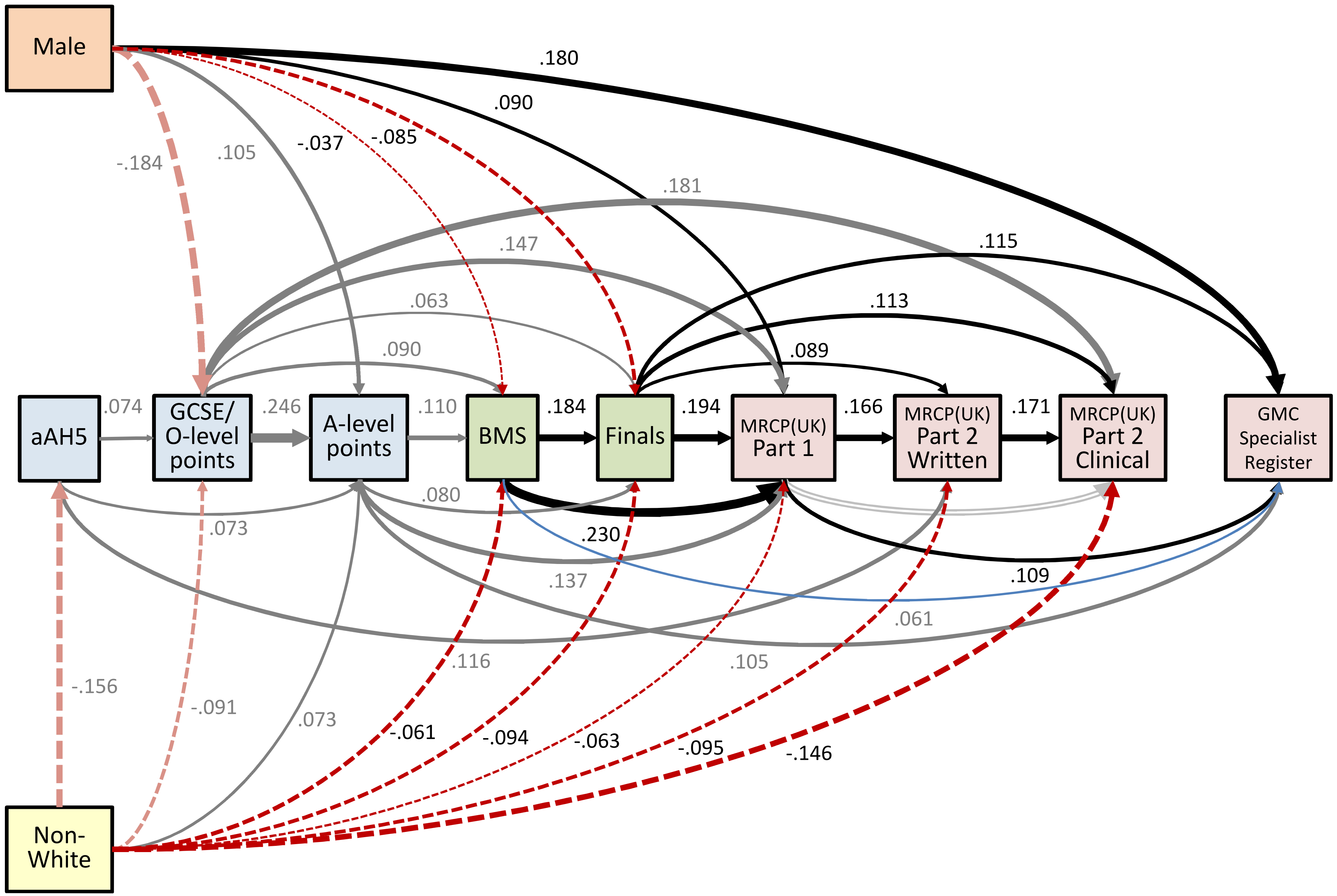

Additional figure 3 (Cohort 85).

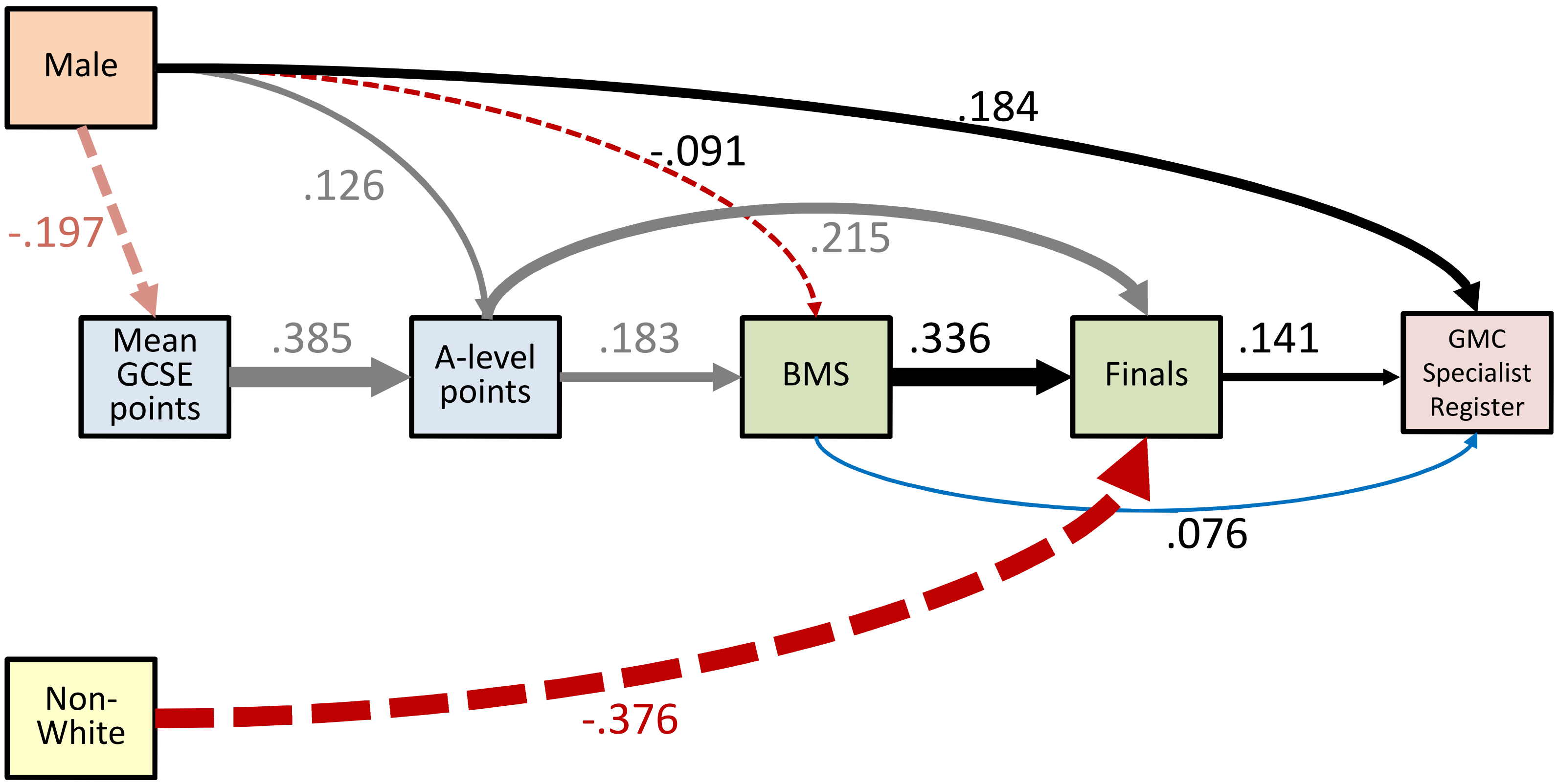

Additional figure 4 (Cohort 80).

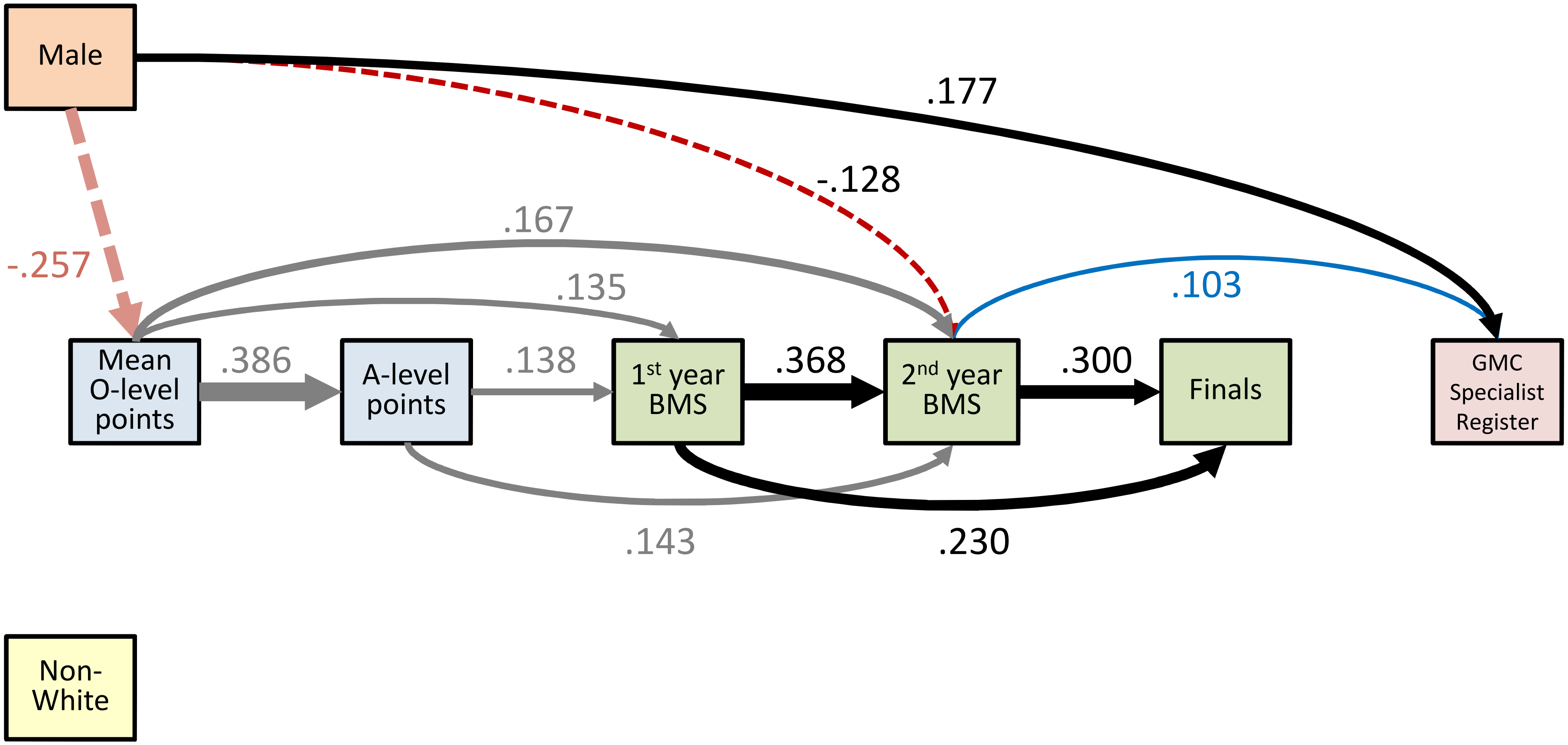

Additional figure 5 (Westminster 1975-82 cohort).

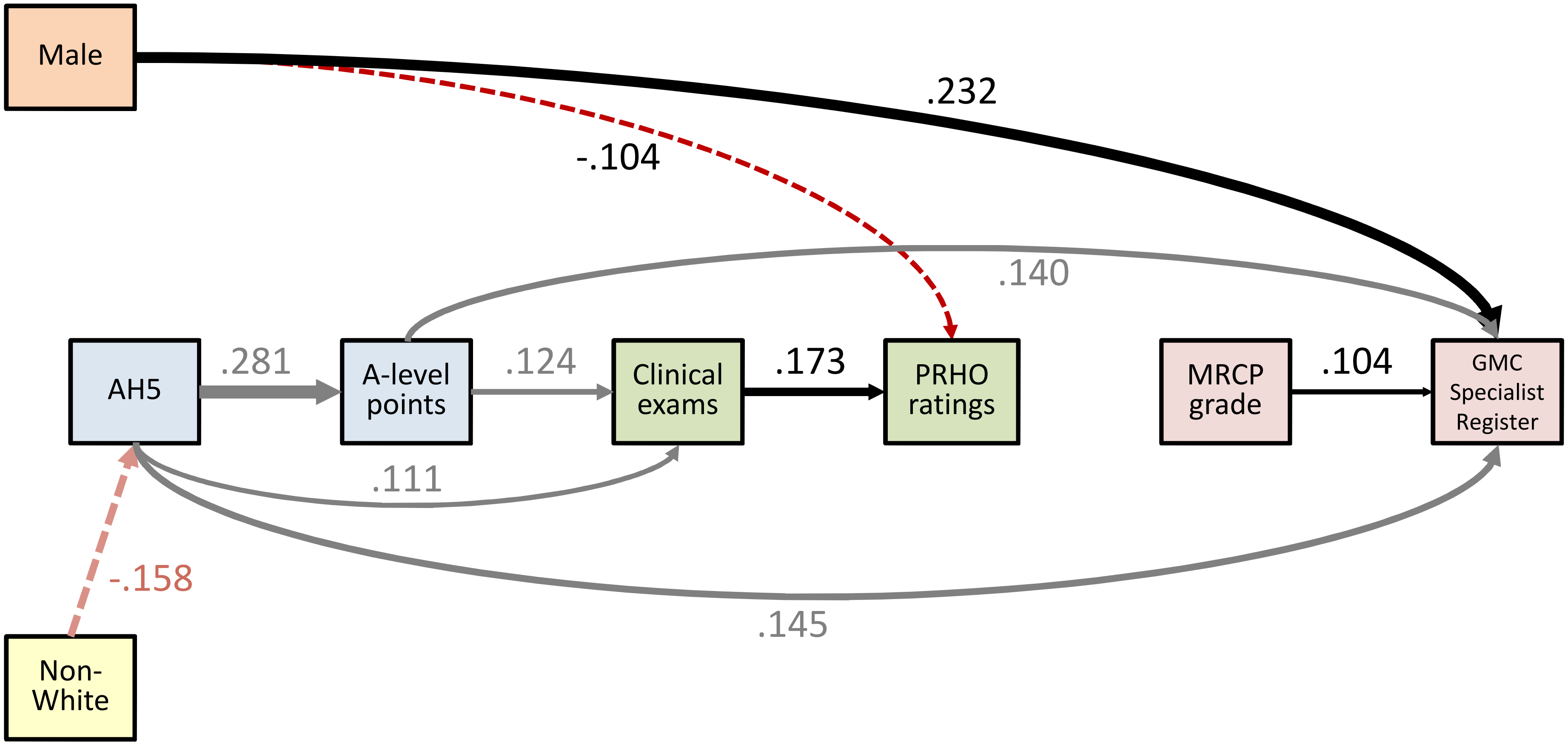

Supplement: Additional file 1 — Sex and ethnicity effects in the five longitudinal cohort studies. [file 1741-7015-11-242-S1.pdf]
