# Supplementary material for: The Academic Backbone: longitudinal continuities in educational achievement from secondary school and medical school to MRCP(UK) and the specialist register in UK medical students and doctors
Source: BMC Med. 2013 Nov 14;11:242. doi: 10.1186/1741-7015-11-242 (PMC3827330; doi:10.1186/1741-7015-11-242)

The MCMC method for correcting correlations for right- and left-censoring

Data such as A-level grades and GCSE grades are strongly right-censored in medical students, many having marks at the ceiling level of AAA for their three best grades. The MCMC algorithm can be used to calculate the mean, standard deviation and the correlation of the true, underlying (latent) distribution.

The method is best demonstrated by synthesising some data with known parameters, censoring it, and then retrieving the true parameters from the censored data. For simplicity the example has a large sample size (10,000), and parameters which are broadly typical of A-level grades. For the two variables, X (horizontally) and Y (vertically) the true means are 30 and 30, the true standard deviations are 3 and 3, and the true correlation is 0.5. Random numbers are grouped into five categories corresponding to scores of 30, 28, 26, 24 and 22 or less on each variable, giving the contingency table shown below.

Score	22 or less	24	26	28	30	*Total*
22 or less	**14**	**18**	**31**	**20**	**14**	*97*
24	**20**	**52**	**111**	**104**	**83**	*370*
26	**30**	**97**	**235**	**336**	**424**	*1122*
28	**20**	**110**	**321**	**603**	**1079**	*2133*
30	**14**	**93**	**398**	**1052**	**4721**	*6278*
Total	*98*	*370*	*1096*	*2115*	*6321*	*10000*

The simple means of the X and Y variables are 28.83 and 28.84, both of which are lower than the correct values of 30 and 30, and the simple SDs are 1.79 and 1.79, both of which are substantially less than the true values of 3 and 3. The empirical correlation of .390 is also less than the true correlation of 0.5.

The MCMC algorithm begins at a starting point (in fact the empirical means, SDs and correlation described in the previous paragraph) and assuming that the distribution is bivariate normal calculates the expected numbers of individuals in each of the cells. As examples, for a cell such as X=24 and Y=28 it uses the *mvncdf* function in *Matlab* to calculate *p*, the expected proportion of a bivariate normal distribution from X=25 to 27 and Y= 27 to 29. The actual frequency of observations in that cell, *f*, which in fact is 110 in the example, is then used to calculates the log.likelihood of the observed *f* individuals in the cell, which is 2.*f*.log(*p*). The likelihood is calculated separately for each cell, cells on the margins having ranges which include Infinity or –Infinity (e.g. for X=22 or less and Y=30 the probability is calculated on the basis of X=-Inf to 23 and Y=29 to +Inf). For the starting values, the summed log.likelihood is 43,662. The MCMC algorithm then alters the various parameters and searches for values which give a lower log-likelihood (and the likelihood for the actual values from which the data were generated is 39,806).

The algorithm is both Monte Carlo and Markov Chain. The Monte Carlo part involves generating estimates of the parameters which are randomly altered at each step of the chain. The Markov Chain part refers to the fact that at step *n+1* the new estimates of the parameters are based on those at step *n* (but not at any previous step). If the log.likelihood is better than at the previous step then the new position is accepted with a fixed probability which is less than one. The result is that the parameters

move through the parameter space, both converging on the best estimates and also particularly sampling the space around those best estimates, eventually ending up in a 'well', which centres around the best fitting values. The parameter space in the current example is actually five dimensional, and figure 1 shows the estimate of each of the parameters at each step in the chain, which is 5000 steps long. Over the first 500 or so links the estimates vary, but after that they become stable, showing random variation around some average point. In the jargon of MCMC, they have become ergodically stable. There are five parameters and therefore it is not possible easily to visualise their joint relationship but figure 2 shows a plot of the values of the mean and standard deviation of X at each of the 5000 steps. The points are coloured in 'jet' colours so that the dark blue point in the lower left-hand corner, indicated with a red arrow, is the starting point and the 'hot' red and yellow colours are at the end of the chain, at the top right. Although the initial points are far removed from the final one, convergence rapidly occurs with estimates localised around 30 for the mean and 3 for the SD.

MCMC allows a straightforward calculation of the standard error and 95% confidence intervals of the various estimates. Considering just the last 2000 steps of the chain, the average estimate of the X mean is 29.96. The standard deviation of the 2000 estimates of X is 0.054, and that can be regarded as an estimate of the standard error of X. The 95 percent confidence intervals can also be calculated by ranking the 2000 estimates, and looking at those at the 2.5th and 97.5th percentiles, which are 29.87 and 30.06. Importantly that interval includes the true value of 30. Similar calculations for the other four parameters gives 95% confidence intervals of 29.94 to 30.13 for the Y mean, 2.89 to 3.05 for X SDF, 2.92 to 3.08 for Y SD, and .473 to .509 for the correlation, all of which include the true value.

If one looks carefully at figure 2 the large 'blob' in the top right-hand corner is not quite circular but is elliptical. That indicates that there is a correlation between the estimates of the mean and SD, and the across the last 2000 steps of the chain the correlation is .399. Parameter estimates often correlate. Essentially what happens in this case is that if the mean is relatively high (say 32), then a reasonable fit can be obtained by also setting the SD higher as well, since all of the fitting process is occurring in the tail of the distribution. Were there only to be two categories (say 28 or less and 30), then it would no longer be possible to estimate both the mean and SD, any value of the mean having an appropriate estimate of the SD which would fit the data perfectly. There would be two parameters but only one degree of freedom, and the correlation between the estimates would necessarily be one.

In this particular case the estimates of the means, SDs and correlation of the decensored distribution are remarkably close to the values in the true distribution from which the numbers were generated, giving faith in the MCMC process in general. Of course the estimates are very good in part because N=10,000, when, for instance the standard error of X mean was .054 and the 95% confidence for the correlation was .473 to .509. If the process is repeated with N=1000, sampling from the same true distribution, then although the average estimate of X mean is 2.91, its standard error is now .148 (and the estimate of the correlation is .446 with 95% confidence intervals of .371 to .520). And if N=100, then although the average estimate of X mean is 29.79, its standard error is now .426 (and the average estimate of the correlation of .568 has 95% confidence intervals of .328 to .738). Standard errors in general are inversely proportional to the square of N, as can be seen by comparing .054 for N=10,000 with .426 for N=100, which are roughly in a ratio of 10:1 (i.e. the square root of 10,000/100). The accuracy of estimates depends therefore on the number of data points in the sample, and *not* the number of elements in the chain.

Figure 1: Plots of the estimated values of the means and SDs of the X and Y values, and their correlations, at each of the 5000 points in the chain. Estimates of the values and their standard errors was based on the last two thousands steps in the chain (indicated by the red box), where the estimates are clearly stable.

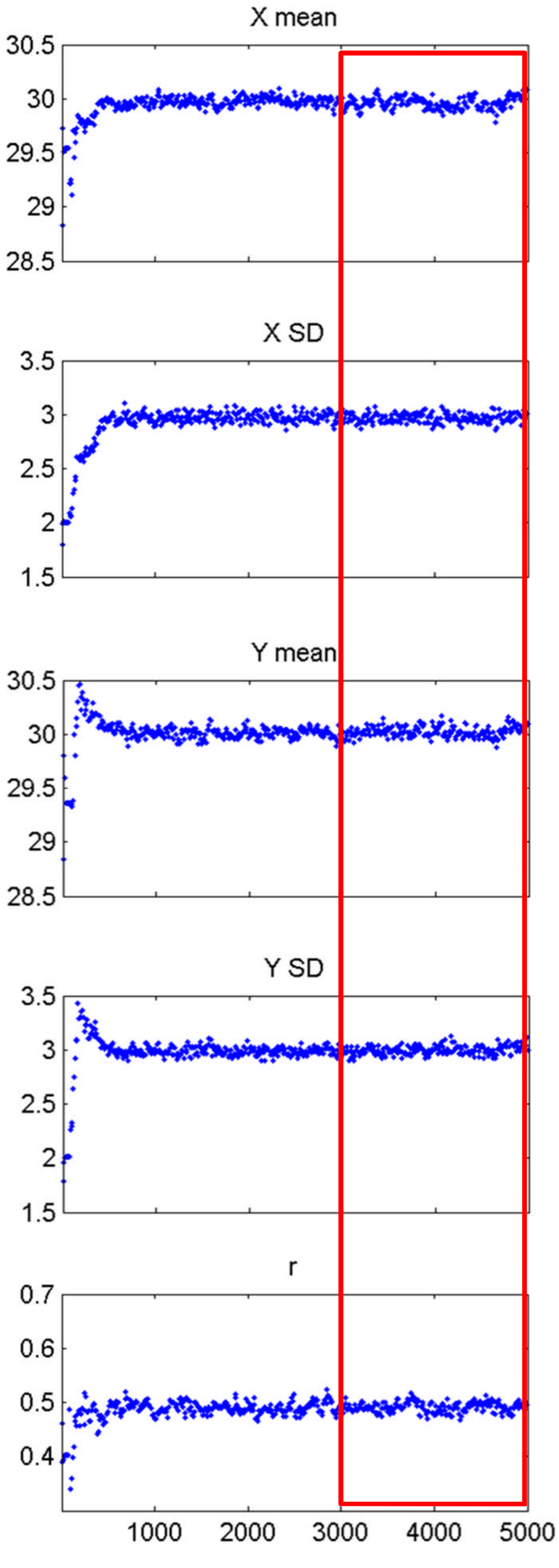

Figure 2: Plots of the estimated values of X mean and X SD at each of the 5000 points in the chain. The red arrow indicates the starting point (at the empirical mean and SD), the red dashed lines the true X mean and X SD from which the data were sampled.

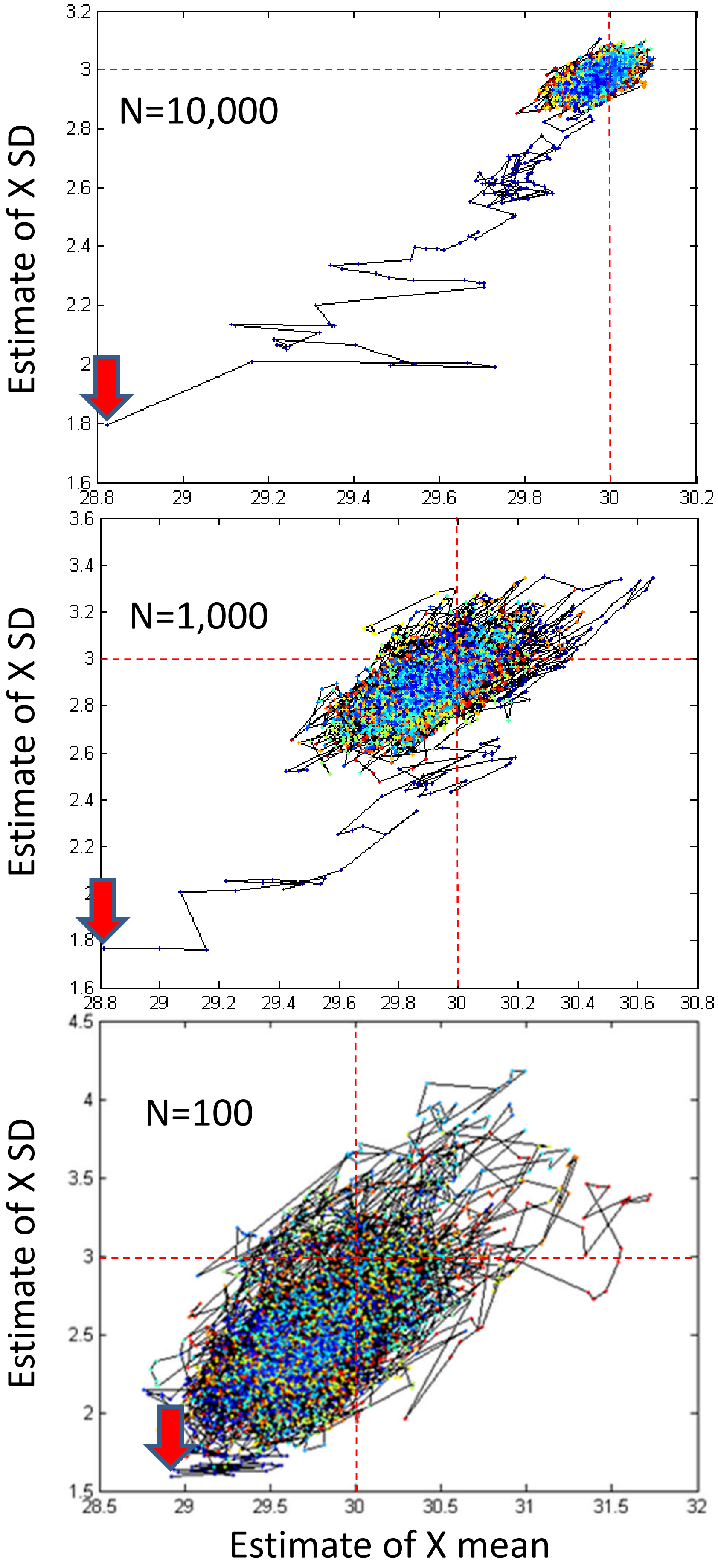

Supplement: Additional file 2 — The MCMC method for correcting correlations for right- and left-censoring. [file 1741-7015-11-242-S2.pdf]
